# FBXW24 controls female meiotic prophase progression by regulating SYCP3 ubiquitination

**DOI:** 10.1002/ctm2.891

**Published:** 2022-07-20

**Authors:** Yang Wang, Wen‐Yi Gao, Li‐Li Wang, Ruo‐Lei Wang, Zhi‐Xia Yang, Fu‐Qiang Luo, Yu‐Hao He, Zi‐Bin Wang, Fu‐Qiang Wang, Qing‐Yuan Sun, Jing Li, Dong Zhang

**Affiliations:** ^1^ State Key Lab of Reproductive Medicine Nanjing Medical University Nanjing China; ^2^ Fertility Preservation Lab and Guangdong‐Hong Kong Metabolism & Reproduction Joint Laboratory Reproductive Medicine Center Guangdong Second Provincial General Hospital Guangzhou China; ^3^ Analysis and Test Center Nanjing Medical University Nanjing China; ^4^ Animal Core Facility Nanjing Medical University Nanjing P. R. China

**Keywords:** FBXW24, meiotic prophase, oocyte, SYCP3, ubiquitination

## Abstract

**Background:**

An impeccable female meiotic prophase is critical for producing a high‐quality oocyte and, ultimately, a healthy newborn. SYCP3 is a key component of the synaptonemal complex regulating meiotic homologous recombination. However, what regulates SYCP3 stability is unknown.

**Methods:**

Fertility assays, follicle counting, meiotic prophase stage (leptotene, zygotene, pachytene and diplotene) analysis and live imaging were employed to examine how FBXW24 knockout (KO) affect female fertility, follicle reserve, oocyte quality, meiotic prophase progression of female germ cells, and meiosis of oocytes. Western blot and immunostaining were used to examined the levels & signals (intensity, foci) of SYCP3 and multiple key DSB indicators & repair proteins (γH2AX, RPA2, p‐CHK2, RAD51, MLH1, HORMAD1, TRIP13) after FBXW24 KO. Co‐IP and immuno‐EM were used to examined the interaction between FBXW24 and SYCP3; Mass spec was used to characterize the ubiquitination sites in SYCP3; In vivo & in vitro ubiquitination assays were utilized to determine the key sites in SYCP3 & FBXW24 for ubiquitination.

**Results:**

*Fbxw24*‐knockout (KO) female mice were infertile due to massive oocyte death upon meiosis entry. *Fbxw24*‐KO oocytes were defective due to elevated DNA double‐strand breaks (DSBs) and inseparable homologous chromosomes. *Fbxw24*‐KO germ cells showed increased SYCP3 levels, delayed prophase progression, increased DSBs, and decreased crossover foci. Next, we found that FBXW24 directly binds and ubiquitinates SYCP3 to regulate its stability. In addition, several key residues important for SYCP3 ubiquitination and FBXW24 ubiquitinating activity were characterized.

**Conclusions:**

We proposed that FBXW24 regulates the timely degradation of SYCP3 to ensure normal crossover and DSB repair during pachytene. *FBXW24*‐KO delayed SYCP3 degradation and DSB repair from pachytene until metaphase II (MII), ultimately causing failure in oocyte maturation, oocyte death, and infertility.

## Graphical abstract

Correct female meiotic prophase is critical for stable genetic passage. Wang et al. identified a novel E3 ubiquitin ligase, FBXW24, which regulated female meiotic prophase progression from pachytene to metaphase II by promoting SYCP3 degradation and double‐strand break repair

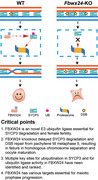

## 1 INTRODUCTION

Meiotic prophase is a delicate process that is crucial for mammalian germ cell survival and reproduction. During this process, homologous chromosomes undergo synapsis which in many organisms is driven by DNA double‐strand break (DSB) repair via homologous recombination. Repair by homologous recombination also produces crossovers that contribute to genetic diversity. These processes correspond to the zygotene and pachytene stages. Afterwards, synapsis between homologous chromosomes is largely removed (diplotene stage), which is the prerequisite for the complete separation of homologous chromosomes during the first meiotic division.^1^, ^2^, ^3^ Errors in any of these processes can cause infertility, sub‐fertility or serious birth defects.^4^, ^5^, ^6^, ^7^, ^8^

Numerous proteins participate in the meiotic prophase. An evolutionarily conserved supramolecular proteinaceous structure termed the synaptonemal complex (SC) is the core player. The SC consists of both central element (CE) and lateral element (LE), as well as, transverse filaments connecting CE proteins to the LEs. The SC begins to assemble on chromosomes during leptotene, zips homologous chromosomes together during pachytene, and is typically gradually disassembled during diplotene. LEs directly interact with chromosomes, and SYCP3 is the key component of LEs.^1^, ^9^, ^10^ Structural studies have demonstrated that the major middle part of the human SYCP3 monomer forms a helical core, and the N‐ and C‐terminal tails are indispensable for the formation of the SYCP3 helical tetramer. Within this tetramer, adjacent monomers are anti‐parallel, and N‐terminal tails bind DNA; together, this structure enables SYCP3 to hold distant regions of DNA together.^11^, ^12^, ^13^, ^14^ In ‐vitro assays show that recombinant SYCP3 can assemble into filamentous fibres with 23 nm repeat units.^12^, ^13^, ^14^ SYCP3 was also required for the strand invasion promoted by the RAD51 and DMC1 recombinases by competing with HOP2‐MND1.^15^ Genetic studies demonstrated that *Sycp3*‐KO embryos developed from oocytes exhibit embryonic death due to chromosome nondisjunction. In addition, DSBs are inefficiently repaired in *Sycp3*‐KO female mouse oocytes.^6^ All of these observations indicate that SYCP3 is essential for homologous recombination.

It has been known that the SC must be disassembled so that homologous chromosomes can be gradually unzipped while remaining connected only at the crossover sites.^1^, ^10^ Activation/inactivation of diverse kinases, such as DDK and CDK1,^16^ MAPK,^17^ PLK1,^18^ and IPL1/AURORA‐B^19^ subsequently targeted the phosphorylation/dephosphorylation of specific SC components such as SYCP2,^17^ SYCP1, and TEX12^18^ or SC regulators such as DBF4^16^ to regulate SC disassembly. Further, ubiquitination/deubiquitination by diverse ubiquitin ligase and deubiquitinases were also found to be important elements.^20^, ^21^ However, as the key component of LEs, the manner in which SYCP3 dynamics are regulated in the meiotic prophase is far from clear. In the present study, we characterized FBXW24 (F‐box and WD‐40 domain protein 24) as an E3 ubiquitin ligase specifically expressed within ovaries and oocytes to regulate the SYCP3 stability, which appears to be essential for meiotic prophase completion and female fertility. Mechanistically, FBXW24 directly ubiquitinates and promotes the timely degradation of SYCP3 in the pachytene, diplotene, and diakinesis stages.

## 2 RESULTS

### 2.1 Oocyte‐predominant FBXW24 is essential for female fertility

In an SKLRB (State Key Lab of Reproductive Medicine)‐independent project, many differentially expressed genes (DEGs) were screened to be the regulators of primordial follicle destiny (in chronological order, germ cells in cysts, germ cells undergoing cyst breakdown, and germ cells in follicles) in PND (post‐natal‐day) 0.5 ovaries through single‐cell RNA sequencing, DEGs with a gradually decreasing expressing level from cyst towards follicles are thought to be more important for those in cysts or even for germ cells in meiotic prophase.^22^ One of these DEGs was *Fbxw24* (gene ID 382106), a gene encoding a protein with both F‐box‐like and WD‐40 domains. The F‐box domain mediates protein‐protein interactions in a variety of contexts, including polyubiquitination,^23^ whereas the WD‐40 domain is proposed to coordinate interactions with other proteins and/or small ligands, playing a wide variety of functions in numerous eukaryotes.^24^

**FIGURE 1:**
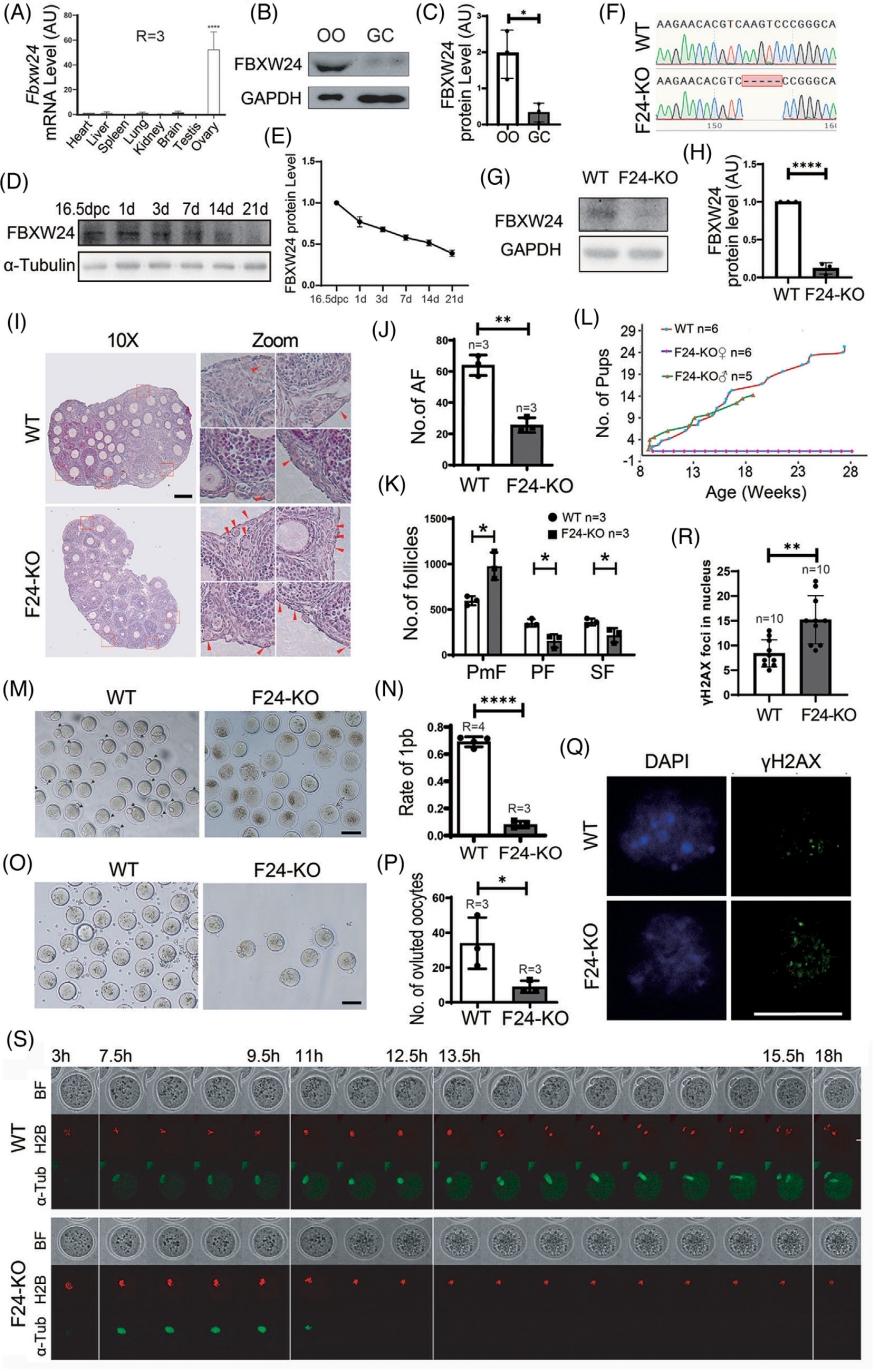
Oocyte‐predominant FBXW24 is essential for female fertility. (A) Q‐PCR shows that *Fbxw24* mRNA was predominant in ovaries. (B and C) Western blots and quantification demonstrate that FBXW24 is more dominant in oocytes than in granular cells (GCs). (D and E) Western blots and quantification show that FBXW24 protein peaked at 16.5 DPC and then gradually decreased from PND 1 to 21. (F–H) Five bases from the third exon of the *Fbxw24* gene were deleted through Cas9 and caused a frame‐shift of the *Fbxw24* gene; western blots show that in *Fbxw24*‐KO ovaries, FBXW24 protein is completely eliminated. (I–K) At PND 21, *Fbxw24* knockout significantly increased primordial follicles (PMF) while it decreased primary follicles (PF), secondary follicles (SF), and antral follicles (AF). Four selected regions (red dot‐line square) from WT and KO ovaries were zoomed and placed on the right, and primordial follicles were arrow‐pointed. (L) Curves for cumulative pups from 2‐month‐old to 7‐month‐old showed that *Fbxw24*‐KO male mice had normal fertility, while *Fbxw24*‐KO female mice were completely infertile. (M and N) *Fbxw24* knockout significantly decreased oocyte maturation rate (first polar body, 1 pb). (O and P) *Fbxw24* knockout significantly decreased numbers of ovulated oocytes. (Q and R) Immunofluorescence and quantification showed that *Fbxw24* knockout significantly increased DSBs (by γH2AX foci) in the GV oocytes. DNA in blue, γH2AX in green. (S) Live imaging shows that oocytes were not able to go through anaphase because homologous chromosomes did not separate at all. Red fluorescence signals are H2B, and green fluorescence signals are α‐Tubulin. Scale bar in Q, 20 μm; Scale bar in other panels, 100 μm. GAPDH was used as a loading control. *, *p* < 0.05; **, *p* < 0.01; ****, *p* < 0.0001. AU, arbitrary unit

We first noted that *Fbxw24* mRNA or protein was predominant in ovaries (Figure 1A and Table S1) and oocytes (Figure 1B,C), and FBXW24 protein decreased gradually from the 16.5 DPC (days post coitum) genital ridge to 21 PND (post‐natal days) in ovaries (Figure 1D,E), indicating that it might be functionally important for meiotic prophase. Upon meiotic prophase resumption (germinal vesicle breakdown, GVBD), FBXW24 sharply decreased in oocytes (Figure S1A,B). FBXW24 was relatively rich within the nucleus at the GV stage, and the nuclear localization was dependent on both microtubules and microfilaments (Figures S1C,D), indicating that its subcellular localization could be affected in various ways. From these preliminary results and the protein domains found in FBXW24, we suspected that FBXW24 may be an E3 ubiquitin ligase working specifically during germ cell meiotic prophase.

Next, we deleted 5 bases from the third exon of the *Fbxw24* gene through Cas9 leading to a subsequent frame‐shift of the *Fbxw24* gene (Figure 1F), which almost completely eliminated the FBXW24 protein (Figure 1G,H). We found that at PND 21 (Figure 1I–K) or 3 (Figures S2A,B), the number of primordial follicles was significantly higher in *Fbxw24*‐KO ovaries compared to the control, whereas follicle numbers at different growing stages significantly decreased. Furthermore, *Fbxw24*‐KO male mice had normal fertility; in contrast, female mice were completely infertile (Figure 1L). Oocyte maturation (first polar body, 1pb, Figure 1M,N) and ovulation (Figure 1O,P) were both sharply reduced. *Fbxw24*‐KO oocytes also had an abnormal metaphase spindle geometry and intensity (Figure S2C–F). Further, significantly increased DSBs indicated by γH2AX staining (Figure 1Q,R) and RPA2 staining (Figure 3A,B) in GV oocytes were noted along with decreased autophagy within GV oocytes (Figure 4A–D), these abnormalities indicated that the oocyte quality was significantly reduced.

Because *Fbxw24*‐KO significantly increased DSBs, we suspected that the chromatin status might be altered as well. We found that *Fbxw24*‐KO significantly decreased meiosis resumption (Figure S5A,B). Most of the KO oocytes had smaller diameters (Figure S5A,C) and immature nuclear configurations (non‐surrounded nucleus, Figure S5D,E). Moreover, *Fbxw24*‐KO oocytes had elevated H2AFY levels (Figures S5F) and increased maternal mRNA (*Gdf9, Bmp15, Zp3*, and *Nobox*) levels (Figure S5G and Table S2). All of these observations indicated that the oocytes were epigenetically immature.^25^, ^26^

Finally, live imaging showed that *Fbxw24*‐KO oocytes were not able to go through anaphase I to metaphase II (MII) as indicated by a complete failure of homologous chromosome separation (Figure S1 and Movie S1).

From these findings, it appeared that *Fbxw24*‐KO caused severe follicle development and oocyte meiosis defects.

### 2.2 *Fbxw24*‐KO delays meiotic prophase progression due to increased SYCP3 level

**FIGURE 2:**
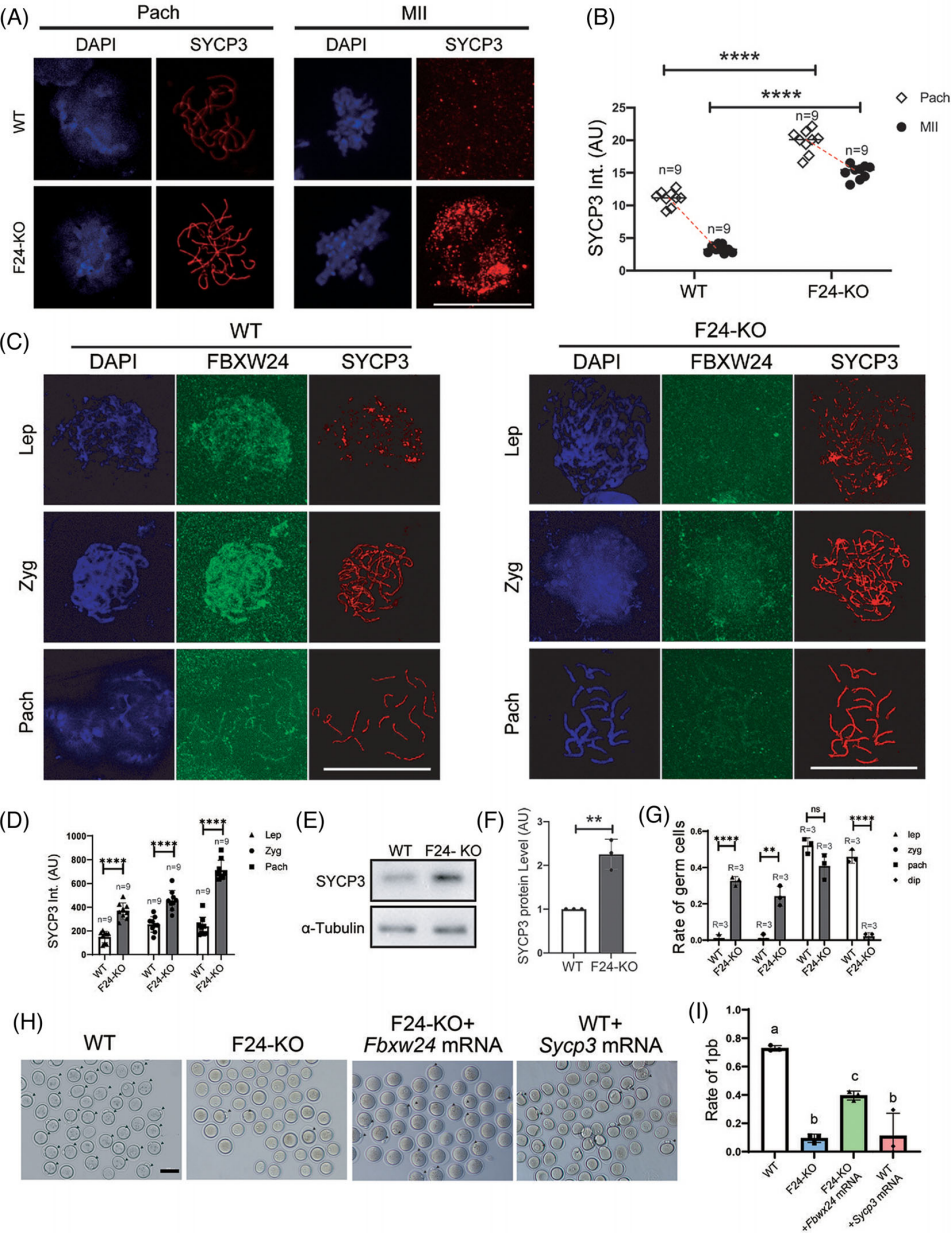
*Fbxw24* knockout delayed meiotic prophase progression due to increased SYCP3 level. (A and B) Immunofluorescence showed that in WT female mice, SYCP3 intensity on spread MII chromosomes was only 20% of the SYCP3 intensity on pachytene chromosomes; while in *Fbxw24*‐KO female mice, SYCP3 intensity on spread MII chromosomes was about 75% of the SYCP3 intensity on pachytene chromosomes. SYCP3 intensity on *Fbxw24*‐KO spread MII chromosomes was about 4‐fold higher than on WT MII chromosomes. DNA in blue, SYCP3 in red. (C and D) Immunofluorescence and quantification showed that *Fbxw24* knockout significantly increased SYCP3 level in meiotic prophase cells at leptotene, zygotene, and pachytene stages within the 16.5 DPC female genital ridge. DNA in blue, FBXW24 in green, SYCP3 in red. "Int." is an abbreviation of "intensity". (E and F) Western blots and quantification showed that the SYCP3 level in *Fbxw24*‐KO 16.5 DPC genital ridge was over a fold higher than in WT. (G) In the WT 19.5 DPC genital ridge, most of the meiotic germ cells are at pachytene or diplotene stages, while in the *Fbxw24*‐KO 19.5 DPC genital ridge, significantly more meiotic germ cells were still at leptotene or zygotene stages. (H and I) Injection of exogenous Sycp3 mRNA into WT GV oocytes significantly decreased oocyte maturation (first polar body extrusion), while injection of exogenous Fbxw24 mRNA into *Fbxw24*‐KO oocytes significantly recovered oocyte maturation. MII oocytes were labelled by black arrowheads. b. Quantification of A. 1pb, first polar body. Scale bar in H, 100 μm; Scale bar in other panels, 20 μm. **, *p* < 0.01; ***, *p* < 0.001; ****, *p* < 0.0001. Different lower‐case letters in I indicate significant differences. AU, arbitrary unit

We next examined whether and how *Fbxw24*‐KO affects meiotic progression. Immunofluorescence showed that in WT female mice, the intensity of SYCP3, which is the SC lateral component directly contacting DNA, was significantly lower on spread MII chromosomes compared to the SYCP3 intensity on pachytene chromosomes; in contrast, in *Fbxw24*‐KO female mice (a small percentage of *Fbxw24*‐KO could survive and progress into MII), SYCP3 intensity on spread MII chromosomes was approximately 75% of the SYCP3 intensity on pachytene chromosomes (Figure 2A,B and Figure S6A,B); These findings implied that SYCP3 has to be degraded for normal progression from meiotic prophase until first meiosis completion, whereas *Fbxw24*‐KO significantly delayed SYCP3 degradation.

Further analysis showed that at each meiotic prophase stage, SYCP3 intensity in *Fbxw24*‐KO genital meiotic cells was significantly higher than in WT cells (Figure 2C,D). Western blots showed that the SYCP3 level in *Fbxw24*‐KO 16.5 DPC genital ridge was more than one‐fold higher than in WT (Figure 2E,F). We further verified the creditability of the SYCP3 increment through immunostaining with another rabbit anti‐SYCP3 antibody (Figure S6A,B) and immunostaining with SYCP3 and centromere (here, centromere intensity was not affected by *Fbxw24*‐KO and served as an internal control, Figure S6C–F). Besides, *Fbxw24‐*KO had no impact on the synapsis at zygotene (Figure S7).

We suspected that the untimely degradation of SC components would impede the unzipping of homologous chromatids at the end of pachytene, thereby impeding meiotic prophase progression. We did find that most of the meiotic cells were still at leptotene or zygotene stages in the *Fbxw24*‐KO genital ridge, even at 19.5 DPC. Comparatively, at this time, most of the meiotic cells in the WT genital ridge were already at pachytene or diplotene stages (Figure 2G). These results, together with the severely reduced oocyte maturation and the failure of chromosome segregation during meiosis, suggested that homologous chromosomes cannot unzip due to the tight “holding” effect of SYCP3. To further support this hypothesis, we injected in vitro transcribed *Sycp3* mRNA into WT GV oocytes and found that oocyte maturation was severely reduced; whereas injection of *Fbxw24* mRNA into *Fbxw24*‐KO oocytes allowed significant oocyte maturation recovery (Figure 2H,I, rate of 1pb, WT vs. F24‐KO vs. F24‐KO + F*bxw24* mRNA vs. WT + S*ycp3* mRNA, 72.8% vs. 9.4% vs. 39.5% vs. 11.1%).

**FIGURE 3:**
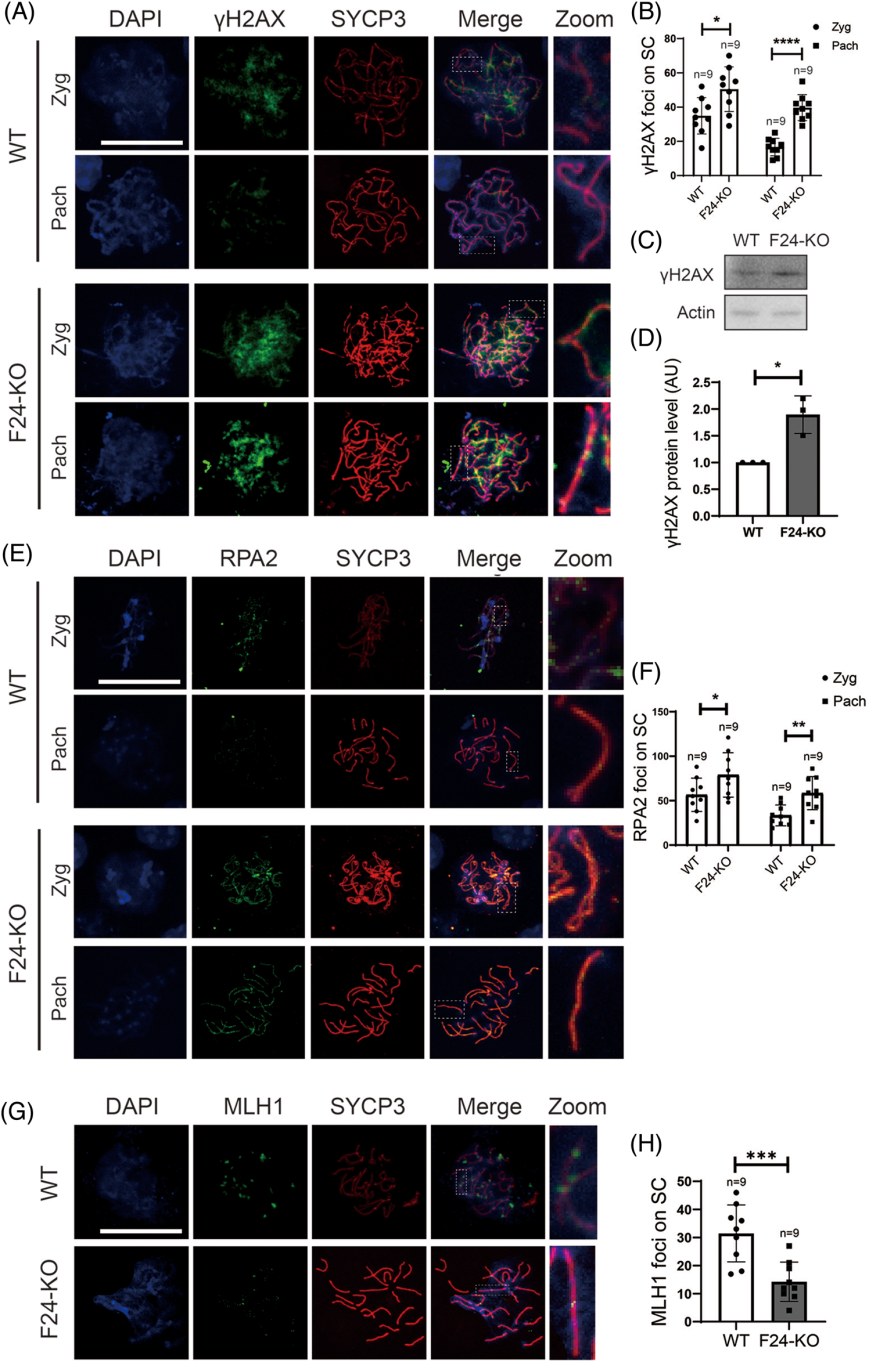
*Fbxw24* knockout increased DSBs and decreased crossover (A and B). Immunofluorescence and quantification showed that the number of γH2AX foci significantly increased in the *Fbxw24*‐KO zygotene or pachytene meiotic germ cells. DNA in blue, γH2AX in green, SYCP3 in red. (C and D) Western blots and quantification showed that the γH2AX level significantly increased in the 16.5 DPC *Fbxw24*‐KO genital ridge. (E and F) Immunofluorescence and quantification showed that the number of RPA2 foci significantly increased in the *Fbxw24*‐KO zygotene or pachytene meiotic germ cells. DNA in blue, RPA2 in green, SYCP3 in red. (G and H) Immunofluorescence and quantification showed that the number of MLH1 foci significantly increased in the *Fbxw24*‐KO pachytene meiotic germ cells. DNA in blue, MLH1 in green, SYCP3 in red. FBXW24 is shortened as "F24" when needed. Actin was used as a loading control. Scale bar, 20 μm. *, *p* < 0.05; ***, *p* < 0.01; ***, *p* < 0.001; ****, *p* < 0.0001. AU, arbitrary unit

At the crossover site, SC has to be disassembled to facilitate recombination, and SYCP3 relocation is essential for SC disassembly.^1^, ^27^ Presumably, SYCP3 hyperstability will impede homologous recombination and DSB repair. We did see significant increments of γH2AX foci in the zygotene and pachytene stages (Figure 3A,B). And western blots also showed that γH2AX levels in the *Fbxw24*‐KO 16.5 DPC genital ridge were significantly higher than in WT (Figure 3C,D). We observed similar changes with another DSB marker RPA2 (Figure 3E,F). Moreover, *Fbxw24*‐KO substantially reduced crossover marked by MLH1 foci (Figure 3G,H), which fits with the hypothesis that elevated Sycp3 during pachytene impedes homologous recombination.

### 2.3 FBXW24 directly ubiquitinates SYCP3

**FIGURE 4:**
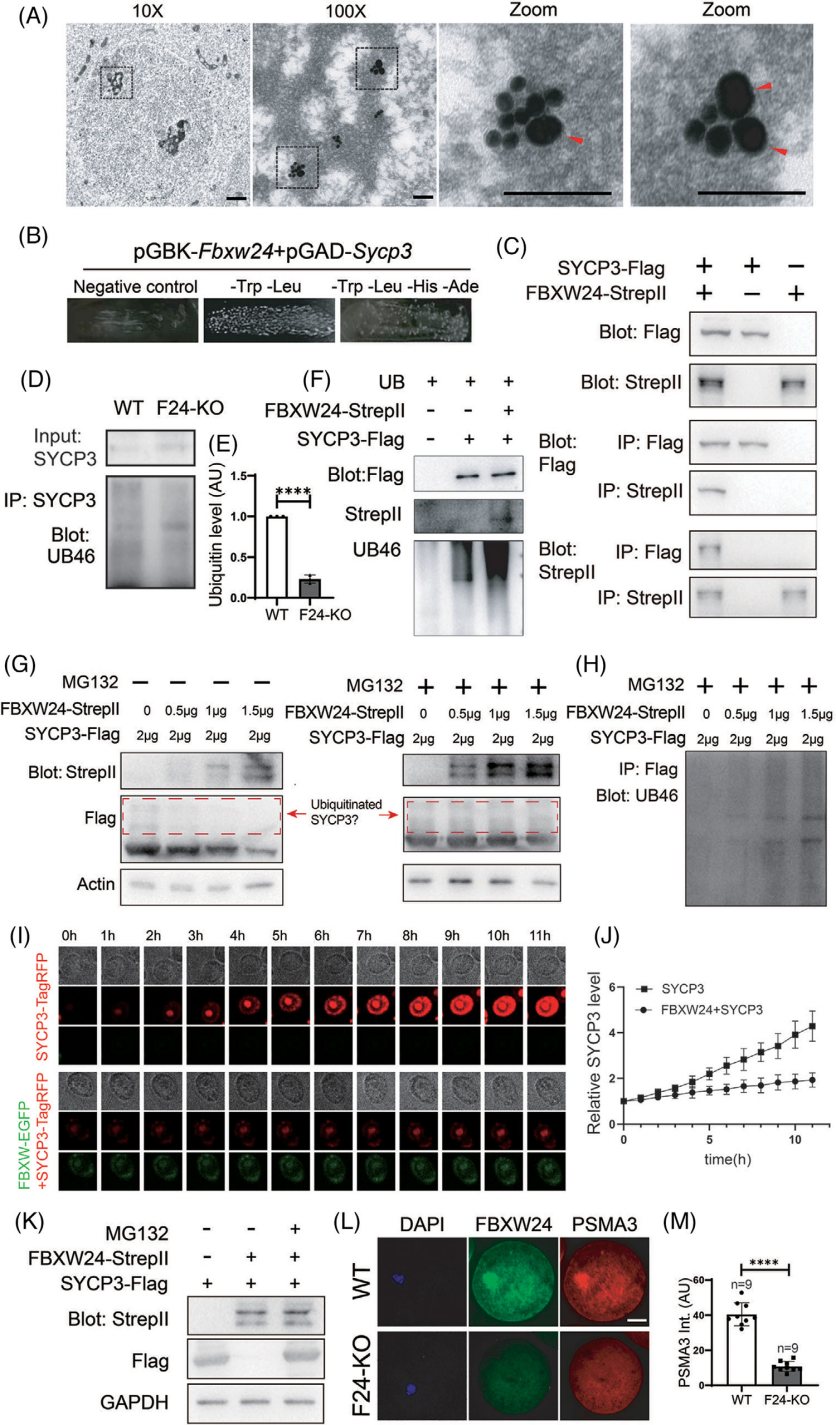
FBXW24 directly binds and ubiquitinates SYCP3. (A) Immuno‐EM shows that in the chromatin regions of meiotic cells in the 16.5 DPC genital ridge, some SYCP3 signals localized closely to FBXW24 (<20 nm). One region (dot‐line square) from a 10x image was photographed at 100x to show the overall signal within the chromatin region; two regions (dot‐line square) from the 100x image were further magnified to show the close adjacency between SYCP3 and FBXW24. SYCP3 and FBXW24 were detected by secondary antibodies conjugated with 15 nm gold (small dots) and 35 nm gold (big dots, arrow‐pointed), respectively. (B) Yeast‐two‐hybridization (Y2H) assay shows that FBXW24 can directly bind SYCP3. (C) Co‐IP between in‐vitro purified FBXW24 and SYCP3 shows that FBXW24 directly binds SYCP3. 1 μg SYCP3‐Flag protein and/or FBXW24‐StrepII protein were/was used in each reaction. (D and E) SYCP3 IP, western blot and quantification demonstrate that *Fbxw24* knockout significantly decreased SYCP3 ubiquitination level. (F) In‐vitro ubiquitination assay showed that in the presence of ubiquitin and other components (Table S5), FBXW24 can significantly increase the SYCP3 ubiquitination level. 1 μg SYCP3‐Flag protein and/or FBXW24‐StrepII protein were/was in each reaction. (G and H) 293T cells were transfected with *Fbxw24*‐StrepII and *Sycp3‐*Flag plasmid (Amounts of plasmid used were in the image), and western blot was done under two conditions. Left of G, in the absence of MG132, as the FBXW24 level increased, the SYCP3 level gradually decreased. Right of G and H, in the presence of MG132, as the FBXW24 level increased, the SYCP3 level remained unchanged, while ubiquitinated SYCP3 gradually increased. Red dot‐line square labelled probable ubiquitinated SYCP3. (I and J) SYCP3 titer were first added into the Sf9 medium to be expressed to a medium level (at about Day 1.5), then FBXW24 titer was added, and SYCP3 intensity almost remained unchanged; In contrast, in the sf9 medium without FBXW24 supplement, SYCP3 intensity keep raising, and big SYCP3 aggregates kept increasing. (K) Western blot shows that FBXW24 expression substantially reduced SYCP3 level; once proteasome activity was inhibited with MG132, SYCP3 level is recovered. (L and M) In WT GV oocytes, PSMA3, a sub‐unit of the proteasome, was enriched within nuclear chromatin and highly overlapped with FBXW24; in *Fbxw24*‐KO GV oocytes, PSMA3 did not show any abundance within chromatin. For all plasmids except B, *Sycp3* is fused to TagRFP and Flag, and *Fbxw24* is fused to EGFP and Strep II. To save space, SYCP3‐TagRFP‐Flag was shortened as "SYCP3‐TagRFP" or "SYCP3‐Flag", and FBXW24‐EGFP‐stepII was shortened as "FBXW24‐EGFP" or "FBXW24‐Strep II" as needed. Scale bar for 10X image in A, 1 μm; for 100X and zoom images in A, 100 nm. Scale bar for other panels, 20 μm. Actin or GAPDH was used as a loading control. ****, *p* < 0.0001. AU, arbitrary unit

Because the KO of a presumed E3 ubiquitin ligase, FBXW24, significantly elevated SYCP3, we suspected that FBXW24 may directly bind and ubiquitinate SYCP3. The following findings supported this direct binding hypothesis: first, immuno‐EM showed that some FBXW24 dots localized close (<20 nm) to SYCP3 dots (Figure 4A) in 16.5 DPC meiotic cells; second, yeast‐2‐hybridization (Y2H) showed that FBXW24 can directly bind SYCP3 (Figure 4B and Figure S8); third, co‐immunoprecipitation between in vitro purified FBXW24 and SYCP3 also showed that FBXW24 directly binds SYCP3 (Figure 4C).

*Fbxw24*‐KO significantly decreased SYCP3 ubiquitination levels (Figure 4D,E). Moreover, in the presence of ubiquitin and other related components (Table S4), in‐vitro purified FBXW24‐EGFP‐strep II protein was able to significantly increase the ubiquitination level of in‐vitro purified SYCP3‐tagRFP‐flag protein (Figure 4F and Figure S9A–C).

In cells, ubiquitinated proteins must be delivered to the proteasome complex for degradation. We verified that as FBXW24 levels increased, SYCP3 levels gradually decreased in 293T cells transfected with FBXW24 and SYCP3 (Figure 4G left). Contrastingly, in the presence of MG132, which prevents the degradation of ubiquitinated proteins without affecting the ubiquitination itself, an increasing level of FBXW24 caused gradually‐increased SYCP3 ubiquitination (Figures 4G right and 4H). Similarly, in Sf9 cells, as the intensity of FBXW24‐EGFP increased, the intensity of SYCP3‐TagRFP accordingly decreased (Figure S10A,B). Besides, when we first added SYCP3 titer expressed to a medium level (at about Day 1.5), then added FBXW24 titer, we saw the SYCP3 intensity increased relatively slowly, in contrast, in the group without FBXW24 supplement, the SYCP3 intensity kept raising, and big SYCP3 aggregates continually increased (Figure 4I,J and Movie S2). Further, once we inhibited proteasome activity with MG132 in 293T cells, the SYCP3 level was recovered (Figure 4K). This finding suggests that SYCP3 degradation relies on both ubiquitination and proteasomes.

In addition, in WT GV oocytes, PSMA3 (a subunit of proteasome) was enriched within nuclear chromatin and highly overlapped with FBXW24; in *Fbxw24*‐KO GV oocytes, PSMA3 did not show any enrichment within chromatin (Figure 4L,M ). Hence, FBXW24 appears to be essential for proteasome recruitment to the SYCP3‐enriched chromatin region.

### 2.4 Characterization of key sites in SYCP3 for ubiquitination

**FIGURE 5:**
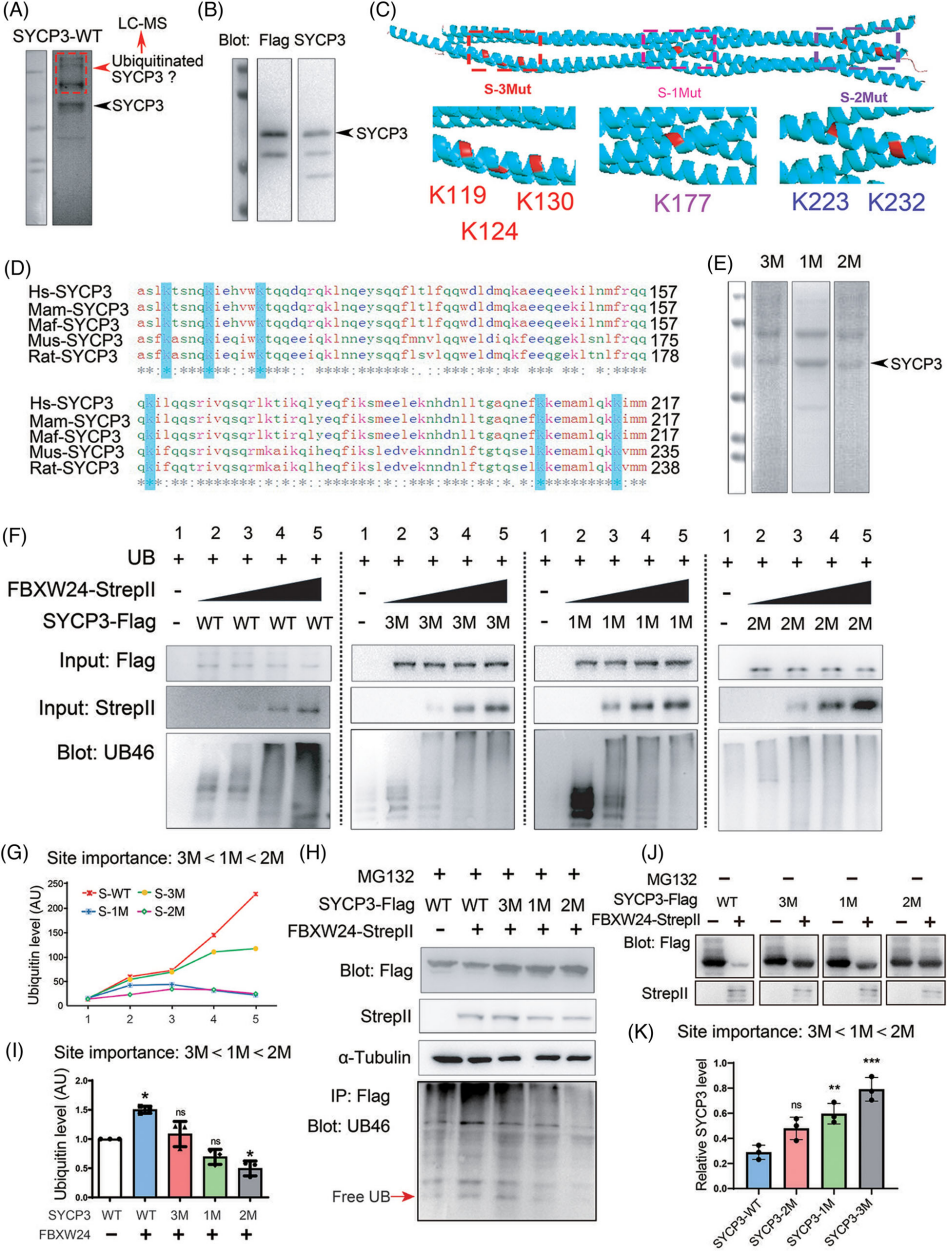
Characterization of key sites in SYCP3 for ubiquitination. (A) Purified WT SYCP3‐TagRFP‐Flag protein was subjected to SDS‐PAGE and Coomassie staining. The stained gel shows that the purified WT SYCP3 demonstrated multiple bands; the lower band (arrow‐pointed) corresponded to the expected size band, and the other upper bands were cut altogether for the characterization of ubiquitination sites within SYCP3 through mass spectrometry (Figure S11A–I and Table S4). (B) The expected right‐size band in A was verified through Flag and SYCP3 western blots (arrow‐pointed). (C) Based on the known SYCP3 structure model, K119, K124, K130, K177, K223, and K232, which are on the surface of the SYCP3 tetramer, were subdivided into three groups according to their positional proximity, and three non‐ubiquitinated mutants were made: K119A, K124A, and K130A – simplified as S‐3 M, K177A – simplified as S‐1 M, and K223A and K232A – simplified as S‐2 M. (D) Protein sequence alignment among SYCP3 from *Homo sapiens* (Hs), *Macaca mulatta* (Mam), *Macaca fascicularis* (Maf), *Mus musculus* (Mus), and *Rattus norvegicus* (Rat) shows that all six selected ubiquitination sites are conserved. (E) SDS‐PAGE and Coomassie staining demonstrate that the purified SYCP3 mutant proteins also showed multiple bands; the right‐size band (arrow‐pointed) is at similar positions to SYCP3 WT protein. (F) In vitro dose‐dependent ubiquitination assay shows that each mutant showed decreased ubiquitination, but the extent of this reduction differed for these three mutants. SYCP3 WT and each mutant reaction were separated by a vertical dot‐line. 2 μg SYCP3 WT or each mutant protein was used in each reaction, 0 μg, 0.5 μg, 1 μg, 1.5 μg, or 2 μg FBXW24 protein was used in each transfection from left to right. (G) Quantification of F shows that the site importance ranking for ubiquitination appeared to be 3 M < 1 M < 2 M. (H and I) In vivo side‐by‐side comparative ubiquitination assay in the presence of MG132 also illustrates that the site importance ranking for ubiquitination appeared to be 3 M < 1 M < 2 M. 2 μg *Sycp3* and/or *Fbxw24* plasmid were/was used in each transfection. (J) In vivo side‐by‐side comparison of FBXW24‐dependent SYCP3 degradation among WT, 3 M, 1 M and 2 M. The degradation extent is also 3 M < 1 M < 2 M. 2 μg *Sycp3* and/or *Fbxw24* plasmid were/was used in each transfection. α‐tubulin was used as a loading control. *, *p* < 0.05; **, *p* < 0.01; ***, *p* < 0.001. AU, arbitrary unit

Next, through mass spectrometry, we characterized eight specific ubiquitination sites within purified SYCP3 (Figure 5A,B, Table S5 and Figure S11A–H). We also used an SYCP3 antibody to perform immunoprecipitation (IP) in 18.5 DPC genital ridges and sent the immunocomplex for mass spectrometry; we found five ubiquitination sites overlapped between these results (Dataset S2, highlighted in blue). Based on the known SYCP3 structure model, we assumed that the sites on the lattice surface of the SYCP3 tetramer may be more accessible for ubiquitination. Therefore, we focused on six conserved sites: K119, K124, K130, K177, K223 and K232, and subdivided them into three groups according to their positional proximity (Figure 5C,D and Movie S3). We made three non‐ubiquitinated mutants: K119A, K124A, and K130A simplified as S‐3 M; K177A simplified as S‐1 M, and K223A and K232A simplified as S‐2 M, and purified these mutants (Figure 5E). We then examined whether these mutants can still be ubiquitinated.

We found that each mutation showed decreased ubiquitination in either the in vitro dose‐dependent (Figure 5F,G) or the in vivo side‐by‐side comparative (Figure 5H,I, with MG132) ubiquitination assay. Further, the extent of the ubiquitination reduction differed for these three mutations: it appeared that K223 and K232 are the most important sites for ubiquitination, whereas K119, K124, and K130 sites are the least important (Figure 5F–I). However, each mutant was still partially degradable by FBXW24, indicating that all these sites contribute to Sycp3 stability (Figure 5J,K, without MG132).

### 2.5 Characterization of key sites in FBXW24 required for SYCP3 ubiquitination

**FIGURE 6:**
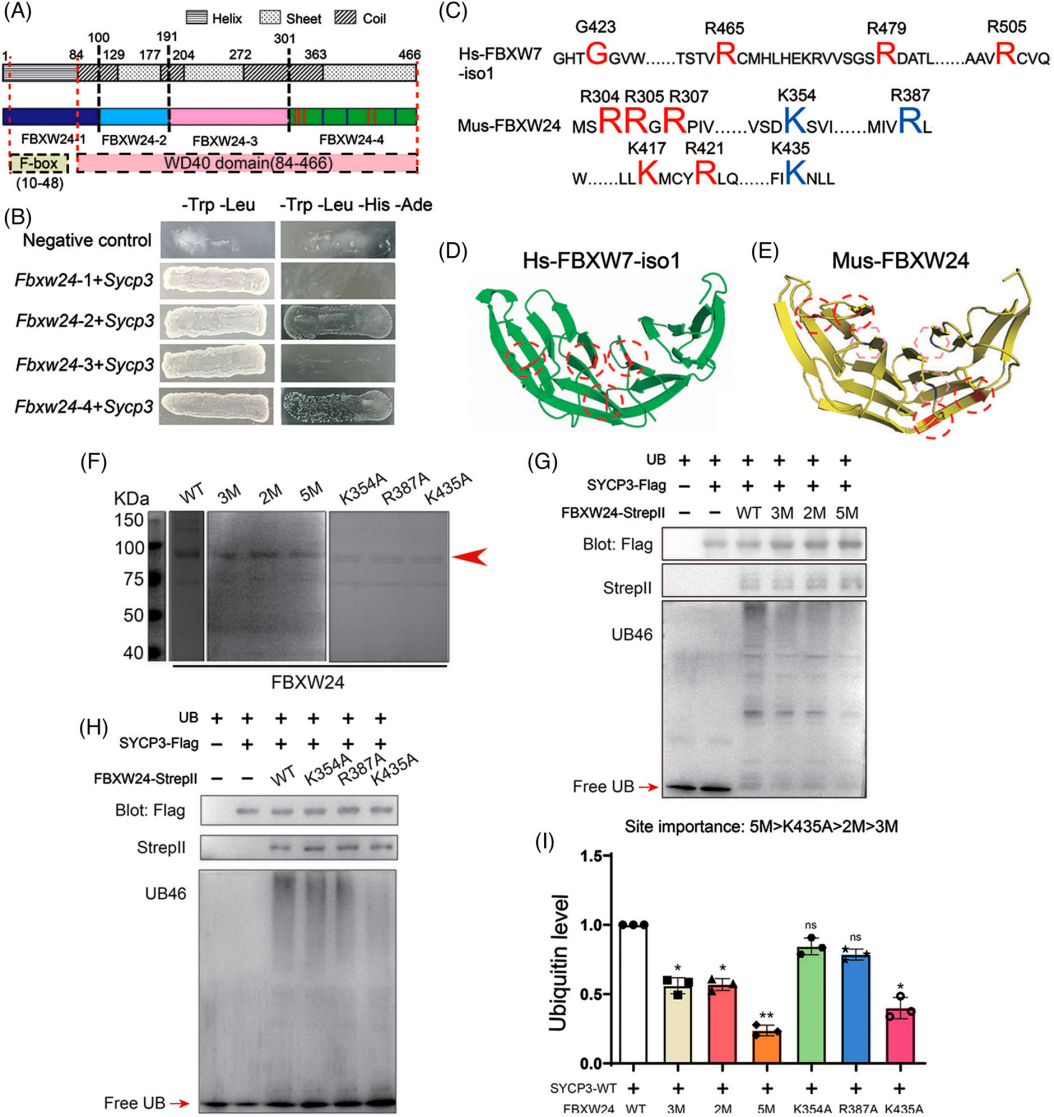
Characterization of key sites in FBXW24 required for SYCP3 ubiquitination. (A) I‐Tasser was used to predict the FBXW24 structure and FBXW24 was sub‐divided into four parts based on its secondary structure. FBXW24‐1 (1–99 AA, dark blue) is primarily α‐helix; FBXW24‐2 (100–191 AA, light blue), FBXW24‐3 (192–300 AA, pink), and FBXW24‐4 (301–466 AA, green) are mostly β‐sheet; and residues between them are primarily coils. Predicted F‐box‐like, from 10–48 AA, and WD40 domain, from 84–486 AA, was also mapped. (B) Y2H showed that FBXW24‐2 or FBXW24‐4 could directly bind SYCP3 through Y2H. (C–E) I‐Tasser showed that the R‐rich C‐terminal region of FBXW24 is structurally similar to the R & K‐rich C‐terminal region of FBXW7. We focused on two groups of R & K‐rich sites (red type in C) within FBXW24‐4 (red dot‐line circles in E) and made three FBXW24 mutants: R304A, R305A, and R307A – simplified as 3 M; K417A and R421A – simplified as 2 M; and R304A, R305A, R307A, K417A, and R421A – simplified as 5 M. We also selected three R & K sites (violet type in C) within FBXW24‐4 (pink dot‐line circles in E) that are spatially similar to the enzymatic sites (red type in C) in the C‐terminal WD domain of human FBXW7‐iso1 (isoform 1, red dot‐line circles in D), and made another three mutants: K354A, R387A, and K435A. (F) SDS‐PAGE and coomassie staining demonstrate the purity of WT, 3 M, 2 M, 5 M, K354A, R387A, and K435A (arrow‐pointed). (G) In vitro side‐by‐side comparative ubiquitination assay shows that all three mutants (3 M, 2 M, and 5 M) have decreased ubiquitinating capacity. 1 μg SYCP3‐Flag protein and/or FBXW24‐StrepII protein were/was used in each reaction. (H) In vitro side‐by‐side comparative ubiquitination assay shows that among K354A, R387A, and K435A, only K435A has significantly decreased ubiquitinating capacity. 1 μg SYCP3‐Flag protein and/or FBXW24‐StrepII protein were/was used in each reaction. (I) Quantification of G and H showed that the site importance is 5 M > K435A > 2 M > 3 M. Different lower‐case letters above the graph column indicate significant differences. Free ubiquitin was arrow‐pointed. AU, arbitrary unit

Next, we attempted to address which sites in FBXW24 are important for its ubiquitination activity. Although we knew from NCBI that FBXW24 is composed of a small N‐terminal F‐box‐like domain (10–48 AA) and a large C‐terminal WD domain (84–486 AA), no studies have yet been conducted on the structure and active residues of FBXW24. We used I‐Tasser for structure prediction and structure‐based function annotation of FBXW24. We sub‐divided FBXW24 into four parts based on its predicted secondary structure. FBXW24‐1 is mostly α‐helix, FBXW24‐2, ‐3, and ‐4 are mostly β‐sheet, and residues between them are primarily coils (Figure 6A, Figure S12A and Movie S4). We next sub‐cloned each part and found that FBXW24‐2 or ‐4 can directly bind SYCP3 through a Y2H assay (Figure 6B), suggesting that the key active residues may be within these two regions. Although not direct evidence, the mutations of diverse arginine residues in human FBXW7 caused an overactivation of multiple kinases involved in cell proliferation, transformation, and metastasis, and arginine and lysine are residues that can undergo diverse post‐translational modifications.^29^ FBXW7 is a well‐known E3 ubiquitin ligase regulating various important proteins.^30^, ^31^ Furthermore, I‐Tasser showed that the R‐rich C‐terminal region of FBXW24 (Figure 6E) is structurally similar to the R‐rich C‐terminal region of FBXW7 (Figure 6D), which belongs to the WD40 repeat motif (Figure 6C–E and Movie S5). Generally, a group of several adjacent charged residues might be the key sites for enzymatic activity. Therefore, we focused on two groups of arginine or lysine sites (Figure 6C, red typo) within FBXW24‐4 (Figure 6E, red dot‐line circle; Figure S12B–F and Movie S5, right model) and made three FBXW24 mutations: R304A, R305A, and R307A – simplified as F‐3 M; K417A and R421A – simplified as F‐2 M; and R304A, R305A, R307A, K417A, and R421A – simplified as F‐5 M. We firstly verified that these mutants have stability similar to FBXW24‐WT (Figure S13A–C) and interact with Sycp3 similarly (Figure S13D). Next, an in vitro side‐by‐side comparative ubiquitination assay showed that all three mutations caused decreased ubiquitinating capacity, while F‐5 M demonstrated the most significant reduction (Figure 6F,G,I), suggesting that all five residues are important for FBXW24's normal ubiquitinating capacity, although their importance rankings differ.

The upper residues, however, are spatially different from those reported as key enzymatic residues in human FBXW7 (highlighted in red, Figure 6C,D, Movie S5, left model). Therefore, we further selected three residues, K354, R387, and K435, which are in similar spatial positions as those reported in human FBXW7 (highlighted in violet, Figure 6C,E; Movie S5, right model) and made three mutants: K354A, R387A, and K435A. We again first verified that these mutants have similar stabilities to FBXW24‐WT (Figure S14A–C) and interact with Sycp3 similarly (Figure S14D). Next, an in vitro side‐by‐side comparative ubiquitination assay showed that only the FBXW24‐K435A mutation caused decreased ubiquitinating capacity (Figure 6H,I), suggesting that only K435 of these three is important for FBXW24's normal ubiquitinating capacity.

These results imply that although mouse FBXW24 shares certain three‐dimensional similarity with human FBXW7 near the C‐terminal WD domain, due to the fairly low conservation of their amino acid sequences (Figure S15), they are largely different from each other in terms of the spatial distribution of enzymatic sites.

### 2.6 *Fbxw24*‐KO increases RAD51 and p‐CHK2 but fails to repair DSBs in female germ cells

**FIGURE 7:**
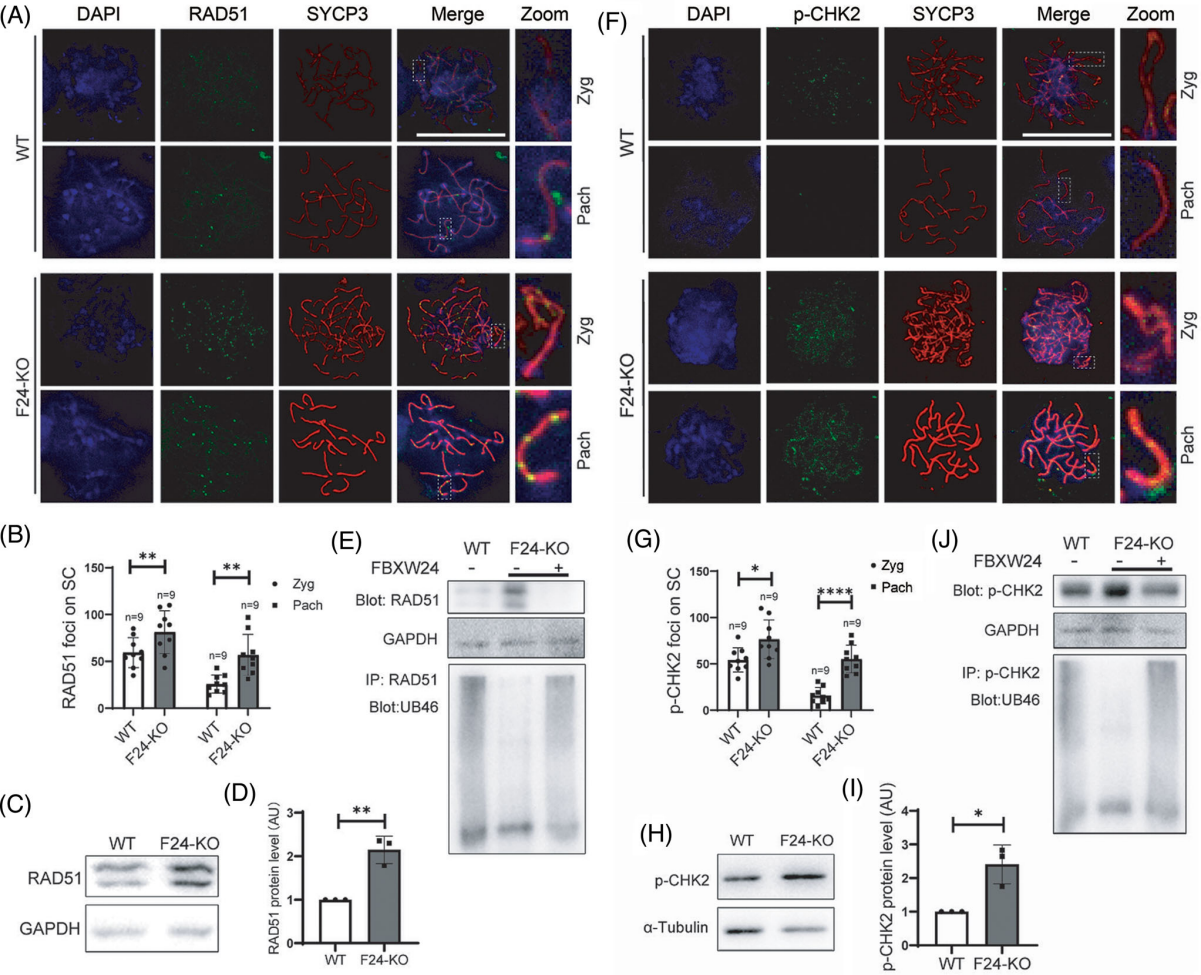
*Fbxw24* knockout increased RAD51 and p‐CHK2 foci in female germ cells. (A and B) Immunofluorescence and quantification showed that RAD51 foci significantly increased in the *Fbxw24*‐KO zygotene or pachytene germ cells. DNA in blue, RAD51 in green, and SYCP3 in red. (C and D) Western blots and quantification show that the RAD51 level significantly increased in the *Fbxw24*‐KO 16.5 DPC genital ridge. (E) RAD51 antibody immunoprecipitation and UB46 western blots demonstrate that RAD51 can be ubiquitinated, while *Fbxw24* knockout substantially reduced RAD51 ubiquitination level. Further, exogenous FBXW24 protein (1 μg) supplementation increased RAD51 ubiquitination level and reduced RAD51 level close to WT. (F and G) Immunofluorescence and quantification showed that the p‐CHK2 foci significantly increased in the *Fbxw24*‐KO zygotene or pachytene germ cells. DNA in blue, p‐CHK2 in green, SYCP3 in red. (H and I) Western blots and quantification show that the p‐CHK2 level significantly increased in the *Fbxw24*‐KO 16.5 DPC genital ridge. (J) p‐CHK2 antibody immunoprecipitation and UB46 western blots demonstrate that RAD51 can be ubiquitinated, while *Fbxw24* knockout substantially reduced p‐CHK2 ubiquitination level. Further, exogenous FBXW24 protein (1μg) supplementation increased p‐CHK2 ubiquitination level and reduced RAD51 level close to WT. α‐tubulin or GAPDH was used as a loading control. *, *p* < 0.05; **, *p* < 0.01. AU, arbitrary unit

Next, we further examined how *Fbxw24*‐KO affects the female meiotic prophase. RAD51 is a key factor in DSB repair, whereas p‐CHK2 monitors and guides germ cells with unrepaired DSBs into apoptosis.^32^, ^33^, ^34^ In *Fbxw24*‐KO meiotic germ cells, RAD51 (Figure 7A–D) and p‐CHK2 (Figure 7F–I) foci both significantly increased. The co‐incidence of unrepaired DSB and increased RAD51 & p‐CHK2 during pachytene appeared counterintuitive, however, *Fbxw24*‐KO caused severe abnormalities that even disabled DSB repair and checkpoint machinery.

Underscoring the connection between FBXW24 and these proteins, we found that these proteins were also subject to ubiquitination, whereas *Fbxw24*‐KO strongly reduced their ubiquitination. Moreover, supplementing the lysate with in vitro purified FBXW24 substantially recovered the ubiquitination (Figure 7E,J). We also examined other essential proteins, including HORMAD1 and TRIP13, but did not find any change (Figure S14A–D), suggesting that FBXW24 could selectively target particular substrates in the meiotic prophase.

### 2.7 *Fbxw24*‐KO affects the level of various chromatin‐related proteins

**FIGURE 8:**
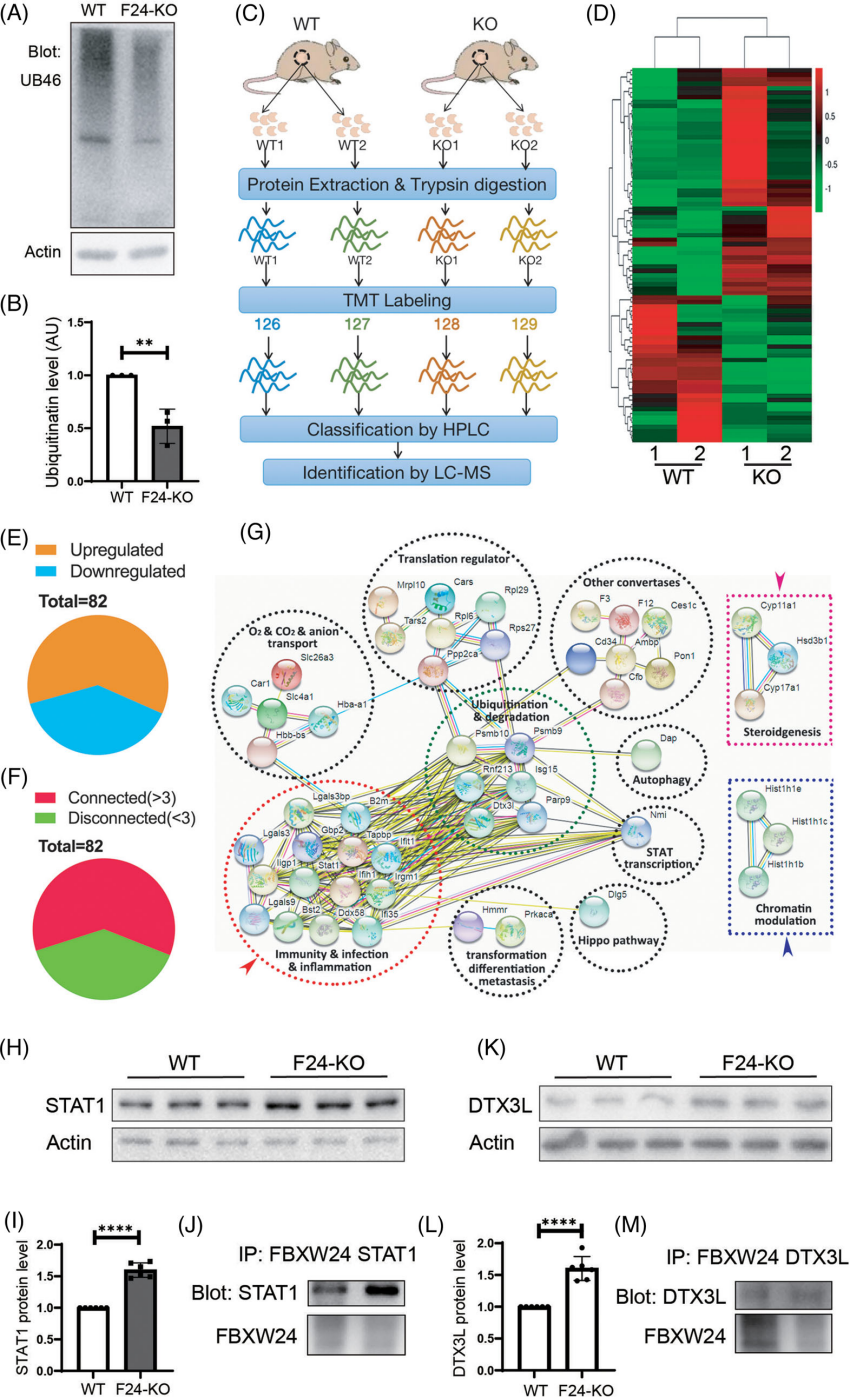
Quantitative proteomics revealed that *Fbxw24* knockout altered the level of various proteins. (A and B) *Fbxw24* knockout substantially reduced pan‐ubiquitination levels in PND 21 ovaries. (C) TMT‐labeled quantitative proteomics was performed to comprehensively investigate the effects of *Fbxw24* knockout on mouse ovaries at the protein level. Peptides from two repeats of WT or *Fbxw24*‐KO ovaries were isobaric‐mass tagged by TMT6‐126, 127, 128, and 129, respectively, and analyzed by LC‐MS. (D) Heat map of 82 differentially expressed proteins (DEPs) at a 1.2‐fold threshold (*Fbxw24*‐KO / WT > 1.2 or < 0.833). (E) Among the 82 DEPs, 60.98% (50 out of 82) were up‐regulated, and 39.02% (32 out of 82) were down‐regulated. (F) By string 2.0, among the 82 DEPs, 60.98% (50 out of 82) connect with each other. (G) KEGG and protein interaction analysis show that six ubiquitination and degradation‐related proteins are in the centre of the interaction network (green dot‐line circle), and around them, there are eight groups of proteins; 14 proteins are involved in immunity, infection, and inflammation processes and consist of the largest group (red dot‐line circle). In particular, there are two separate groups: one group containing Cyp11a1, Cyp17a1, and Hsd3b1, all of which are involved in steroidogenesis (pink dot‐line rectangle); and the other contains Hist1h1b, Hist1h1c, and Hist1h1e, which are all involved in chromatin modulation (blue dot‐line rectangle). (H and I) Western blot verified that *Fbxw24* knockout increased STAT1 protein level. (J) Co‐IP and western blot showed that FBXW24 interacted with STAT1. (K and L) Western blot verified that *Fbxw24* knockout increased DTX3L protein level. (M) Co‐IP and western blot showed that FBXW24 interacted with DTX3L. Actin was used as a loading control. *, *p* < 0.05. AU, arbitrary unit

*Fbxw24*‐KO significantly reduced the pan‐ubiquitination levels in PND 21 ovaries (Figure 8A,B). To further determine the overall mechanism regarding how FBXW24 works, we performed TMT‐labeled quantitative proteomics to comprehensively understand the effects of *Fbxw24*‐KO on mouse PND 21 ovaries at the protein level (we choose post‐natal ovaries rather than genital ridge for this task due to the extremely low weight of the genital ridge sample) (Figure 8C). At the threshold of *Fbxw24*‐KO / WT > 1.2 or < 0.833, there were 82 DEPs (differentially expressed proteins) (Figure 8D), and among them, 60.98% (50 out of 82) were up‐regulated, and 39.02% (32 out of 82) were down‐regulated (Figure 8E). By string 2.0, we found that 60.98% (50 out of 82) were connected (number of connecting members, ≥ 3) (Figure 8F). KEGG and protein interaction analysis showed that six ubiquitination and degradation‐related proteins are in the centre of the interaction network, and there are eight groups of proteins around them (Figure 8G). Fourteen proteins are involved in immunity, infection, and inflammation processes and comprise the largest group. In particular, there are two separate groups, one containing Cyp11a1, Cyp17a1, and Hsd3b1 – all of which are involved in steroidogenesis – and the other containing Hist1h1b, Hist1h1c, and Hist1h1e – all of which are involved in chromatin modulation. The diversity and importance of these pathways further indicate that FBXW24 targets many essential substrates and participates in diverse and important pathways. We selected two DEPs, STAT1 and DTX3L, which could respond to DNA damage and regulate DNA repair,^35^, ^36^ from the top 15 upregulated proteins and verified the upregulation by western blot (STAT1, Figure 8H,I; DTX3L, Figure 8K,L). We also verified that either protein interacted with FBXW24 (Figure 8J,M).

## 3 DISCUSSION

In this study, we for the first time, found that a novel mouse E3 Ubiquitin ligase that is exclusively expressed within mouse oocytes and ovaries, FBXW24, is essential for female fertility. From all of our obtained evidence, we propose that FBXW24 promotes meiotic prophase progression by promoting the timely degradation of various meiotic prophase‐regulating proteins, one of which is SYCP3. *Fbxw24‐*KO impeded SYCP3 degradation during pachytene, delayed meiotic prophase progression, increased DSB and decreased crossover. Moreover, non‐degraded SYCP3 and unrepaired DSB persisted until MII (meiosis II), which ultimately led to failure in homologous chromosome segregation and oocyte death during meiotic maturation. Furthermore, we characterized and ranked multiple key sites in SYCP3 essential for ubiquitination and sites in FBXW24 essential for ubiquitin ligase activity. However, *Fbxw24*‐KO seemed to cause a more complicated disorder: *Fbxw24*‐KO significantly increased p‐CHK2, a change that should be able to eliminate defective germ cells, however, this elimination did not occur; *Fbxw24*‐KO also significantly increased Rad51 that should be able to repair DSB, however, this repair did not occur either. All these findings indicate that FBXW24 most likely also regulates other important targets.

Post‐translational modifications of various SC components have been shown to regulate the SC disassembly during the late meiotic prophase. For example, in *C. elegans*, inactivation (dephosphorylation) of MAP kinase at late pachytene is critical for the timely disassembly of the SC proteins from the long arms of the bivalents, whereas constitutive phosphorylation of SYP‐2 by MAPK at S25 prevents the disassembly of the SC proteins.^17^ In pachytene mouse spermatocytes, only PLK1 of the PLKs is localized to the SC. PLK1 inhibition prevented the phosphorylation of CE proteins SYCP1 and TEX12 and their removal from SC; these changes delayed prophase exit.^18^ However, phosphorylation itself does not lead to SC component degradation. Interestingly, in *Drosophila* oocytes, the correct assembly of SC components requires the E3 ubiquitin ligase SINA. Mutation in *Sina* caused SC components to assemble into large, rod‐like polycomplexes instead of the correct zipper‐like structure, suggesting that Sina protein inhibits the polymerization of SC components; however, which SC components were ubiquitinated and subsequently degraded was not addressed.^20^ SYCP3 is one of the key components of LEs, and it was found that the level of SYCP3 dramatically became reduced after pachytene.^37^ Hence, SYCP3 degradation may be essential for prophase progression.

In the present study, we, for the first time, found that FBXW24‐KO significantly increased SYCP3 level and delayed prophase progression; FBXW24 directly binds and ubiquitinates SYCP3 in vivo and in vitro; and the ability of FBXW24 to reduce SYCP3 can be inhibited by MG132, a proteasome inhibitor. All of these observations suggest that degradation of SYCP3 by FBXW24 through ubiquitination promotes prophase progression. Although there has been speculation that SYCP2 and SYCP3 are not required for centromere cohesion at the first meiotic division,^38^ it was not known how increased levels of the SYCP3 protein affect meiosis; we found that FBXW24‐KO dramatically increased the SYCP3 signal at the meiotic centromere and led to massive oocyte death during meiotic maturation (MII). Moreover, exogenous *Sycp3* mRNA injection partially recapitulates the maturation failure of *Fbxw24*‐KO GV oocytes, while exogenous *Fbxw24* mRNA injection can partially but significantly recover the oocyte maturation rate of *Fbxw24*‐KO oocytes. These findings suggest that an abnormally high SYCP3 level not only delays meiotic prophase but also impedes meiotic maturation.

We also, for the first time, identified six key sites in SYCP3 required for ubiquitination, and we determined their relative importance ranking. K223 and K232 – which localize close to the c‐terminal – appeared to be the most important sites, potentially because ubiquitination of these two sites can efficiently disassemble the SYCP3 tetramer due to the N‐ and C‐terminal tails being indispensable for the formation of the SYCP3 helical tetramer.^11^, ^12^, ^13^, ^14^

Besides SYCP3, we also found that FBXW24‐KO significantly elevated RAD51 and p‐CHK2 levels. Accordingly, we determined that FBXW24‐KO decreased their ubiquitination level, whereas FBXW24 supplementation resulted in the ubiquitination level recovery that was at the control levels. This result suggests that FBXW24 can ubiquitinate other important prophase proteins as well. Notably, in somatic cells, ubiquitination of Rad51 by E3 ubiquitin ligase RFWD3 promoted RAD51 foci turnover and the timely degradation of RAD51 to allow HR progression to subsequent steps.^39^ FBH1 (F‐box DNA helicase 1) is the only known 3′–5′ DNA helicase with F‐box, and it also ubiquitinates RAD51 and negatively regulates RAD51 function,^40^ but, RFWD3 and FBH1 are not part of the FBXW24 family. In *C. elegans* meiosis, BRCA1‐BARD1 forms an E3 ubiquitin ligase to regulate the localization and level of RAD51; however, whether or not RAD51 is ubiquitinated was not examined.^41^ Thus, it appears that FBXW24 might also regulate the turnover of various other important proteins during the female meiotic prophase.

FBXW24 belongs to a super protein family, F‐box. However, the conserved F‐box is a particularly short motif (approximately 40 amino acids), and there is a relatively large difference between F‐box members beyond this motif that leads to distinct members functioning in a highly distinctive manner. For example, FBXO47 is predominant in the testis while FBXO47 prevents TRF2 degradation as a ubiquitination inhibitor during male meiosis and maintains initial telomere attachment to the inner nuclear envelope, and Fbxo47‐deficient spermatocytes are unable to form a complete SC.^42^ During mouse oocyte meiosis, FBXO30 ubiquitinates target stem‐loop‐binding protein (SLBP) for degradation through ubiquitination. FBXO30 depletion elevated the SLBP level; leading to excessive histone H3 on chromosomes and inhibited chromosome segregation.^43^ We cannot determine the human homolog of FBXW24 based on homology. Through a combination of evidence, it appears that R304, R305, R307, K417, R421, and K435 are critical for its ubiquitin ligase activity. Interestingly, in ECs, 30% of serious human endometrial cancers include somatic mutations in FBXW7. These mutations are mostly arginine residues near the C‐terminal of the WD domain.^29^ The upper key residues in mouse FBXW24 are also localized at the C‐terminal of the WD domain; however, the detailed spatial position of these key enzymatic residues between human FBXW7 and mouse FBXW24 significantly differs. Further investigations are required to determine whether there is and, if so, which is the human homolog of mouse FBXW24.

**FIGURE 9:**
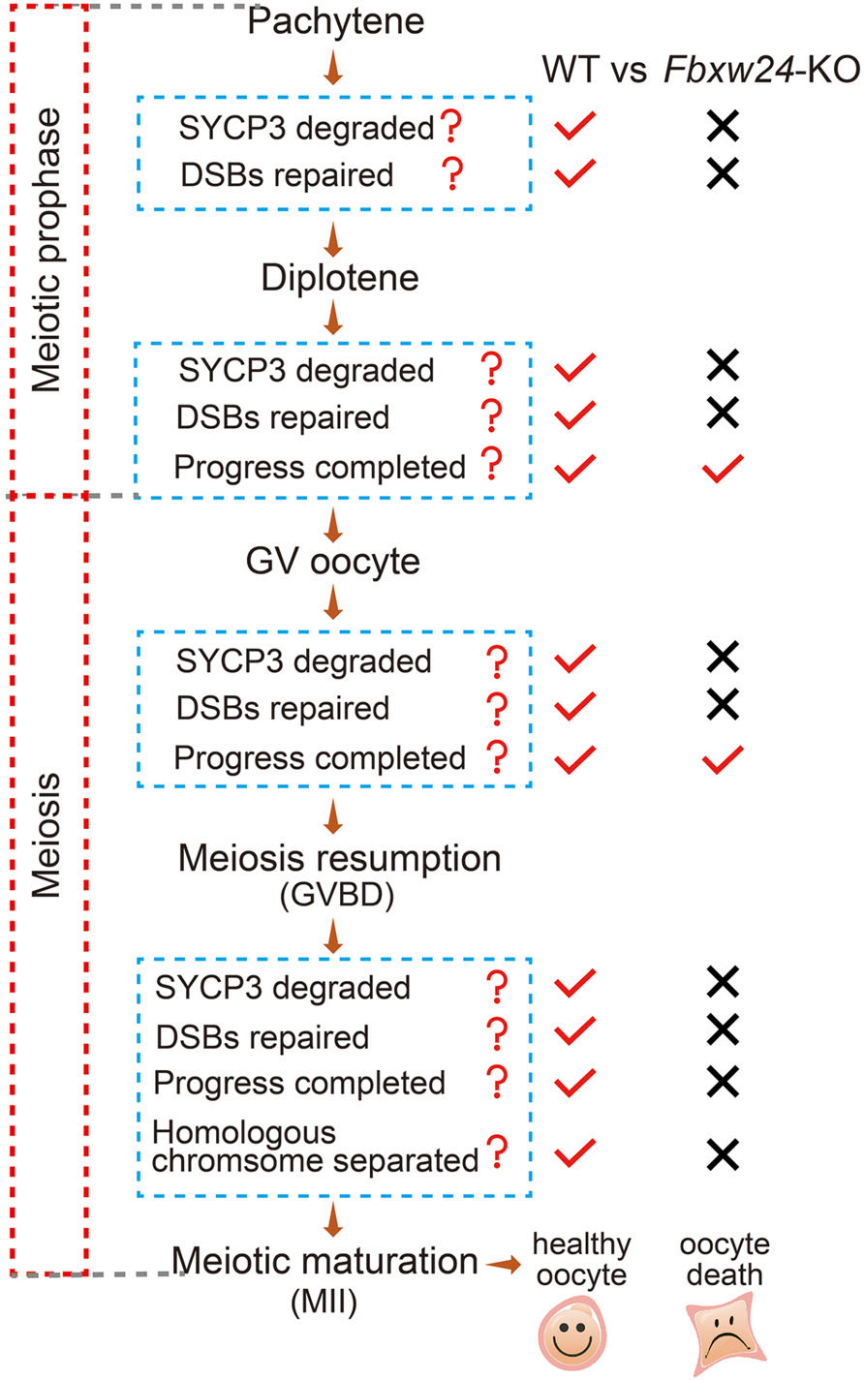
FBXW24 working model. This model showed the correlation between FBXW24, SYCP3 degraded, DSB repaired, and meiotic progression from pachytene to MII. In WT female germ cells, an average level of FBXW24 promotes SYCP3 degradation during pachytene, which subsequently promotes DSB repair. Therefore, germ cells can keep progressing till MII. In *Fbxw24‐*KO female germ cells, *Fbxw24* knockout delayed SYCP3 degradation, subsequently, DSB cannot be repaired, pachytene progression is delayed; delayed Sycp3 degradation and unrepaired DSB remained until MII, which conduced to non‐separated homologous chromosomes and unrepaired DSB, ultimately resulted in MII oocyte death

In summary, we proposed that FBXW24 regulates the timely degradation of SYCP3 to ensure normal DSB repair and meiotic prophase progression. *Fbxw24*‐KO caused delayed SYCP3 degradation and unrepaired DSB from pachytene until MII, ultimately leading to defective oocyte maturation and infertility (Figure 9). Further investigations are required to uncover how FBXW24 regulate other proteins essential for meiotic prophase. This study will make an important contribution to the mechanism of meiotic prophase and might be a new reference for clinical female reproductive diseases.

## 4 MATERIALS AND METHODS

### 4.1 Animal models

CRISPR/Cas9 technology was used to generate the global Fbxw24‐KO mice. 20‐base template sequence of the sgRNA, 5′ CTCTTTGCCCAGGAGCTGTA3’, was in the third exon of the Fbxw24 gene and inserted into pUC57‐T7‐gRNA. MEGAshortscript Kit (Thermo) was used to produce sgRNA from a linearized pUC57‐T7‐Fbxw24‐gRNA template. SgRNA was then purified by MEGAclear Kit (Thermo). mMessage mMachine T7 kit (Thermo) was used to produce cas9 mRNA, from linearized pST1374‐N‐NLS‐flag‐linker‐Cas9. Poly A‐tailing kit (Thermo) was used to poly A‐tailed mRNA to increase its stability. mRNA was purified from the RNeasy Micro kit (Qiagen, Düsseldorf, Germany). SgRNA and cas9 mRNA were assigned to the Animal Core Facility (ACF) of Nanjing Medical University, where a professional ACF staff did pronuclear microinjection, embryo transfer and mouse parturition. Then we genotyped the Fbxw24‐KO mice with the following primers: Forward primer is 5′‐GCACACTGTAACCATGGCTC‐3′; reverse primer is 5′‐AGAATGTAGCACTGCACAGAATG‐3′.

The Institutional Animal Care and Use Committee (IACUC) of Nanjing Medical University approved our experimental animal procedures (Approval No: IACUC‐1809011 and IACUC‐1810013). All mice were housed under standard specific pathogen‐free (SPF) conditions of ACF.

### 4.2 Antibodies

Primary antibodies: mouse anti‐GAPDH (Cat#: 30201ES60; YEASEN, China); mouse anti‐β‐Actin (Cat#: A5316‐100; Sigma, USA); mouse anti‐ace‐Tubulin (Cat#: T‐7451; Santa Cruz, USA); human anti‐Centromere crest (Cat#: 15–234; Antibodies Incorporated, USA); mouse monoclonal anti‐SYCP3 (Cat#: ab97672; Abcam, UK); rabbit polyclonal anti‐SYCP3 (Cat#: ab15093; Abcam); mouse anti Strep II Tag (Cat#: YFMA0054, Yifeixue, China); mouse anti Flag Tag (Cat#: D190828, BBI Life Science, China); rabbit anti histone γH2AX(S139) (Cat#: AF3187, Affinity, USA); rabbit anti H2AFY (Cat#: BS8670; Bioworld, USA); rabbit polyclonal anti histone H3 (Tri‐Methyl K4) (Cat#: BS8670; Bioworld, USA); rabbit polyclonal anti histone H3 (Tri‐Methyl K27) (Cat#: BS7237; Bioworld, USA); rabbit polyclonal anti phosphorylated CHK2 (Thr68) (Cat#: AF3036; Affinity, USA); rabbit polyclonal anti Ubiquitin (A46) (Cat#: BS1487; Bioworld, USA); rabbit polyclonal anti RAD51 (Cat#: BS91146; Bioworld, USA); rabbit anti PSMA3 (Cat#: 11887‐1‐AP; Proteintech, China); rabbit anti LC3b (Cat#: A5205; Bimake, USA). Rabbit polyclonal anti‐FBXW24 antibody was made by Willget Biotech (Shanghai, China) and purified by antigen affinity purification, the antigen sequence is VASFSLQDYNENPK. Antibody specificity was verified by siRNA knockdown and western blot (Figure S17A–L and Table S6).

Secondary antibodies: horseradish peroxidase (HRP)‐conjugated rabbit anti‐goat IgG and HRP‐conjugated goat anti‐mouse IgG were purchased from Vazyme (Nanjing, Jiangsu, China). Cy2‐conjugated donkey anti‐mouse IgG (Code: 715‐225‐150), Rhodamine (TRITC)‐conjugated donkey anti‐human IgG (Code: 709‐025‐149), Cy2‐conjugated donkey anti‐human IgG (Code: 709‐225‐149), and Cy2‐conjugated donkey anti‐rabbit IgG (Code: 711‐225‐152), were purchased from Jackson ImmunoResearch Laboratory (West Grove, PA, USA).

### 4.3 Immunofluorescence staining of female germ cells

Genital ridges were isolated from 16.5 or 19.5 DPC (day‐post‐cross) female mice in operation medium (Hepes). Tissues were mechanically dispersed by syringe needles into 1 mg/ml collagenase in phosphate buffer solution (PBS) at 37.0°C for several hours until the tissues were digested into single cells. Then, the cells were incubated at 37.0°C for 1.5 h with HEB (Hypo extraction buffer; 0.17 M trisodium citrate dehydrate, 0.1 M PMSF, 0.6 M Tris, 0.5 M EDTA, 0.5 M Sucrose, 0.5 M DTT) and transferred onto an adhesive slide, then permeated and fixed with 1% paraformaldehyde (PFA)/0.25% Triton X‐100 at 4.0°C overnight. Samples were washed for 10 min with PBST (0.1% Triton‐X‐100 in PBS) and subjected to a blocking buffer (5% BSA in PBS) for 30 min to block non‐specific antibody binding. After that, they were incubated with primary antibody diluted in blocking buffer overnight at 4.0°C, washed three times (10 min each) in PBS, incubated with secondary antibody diluted in blocking buffer at 25.0°C for 45 min, and washed three times (10 min each) in PBS. Finally, 3 μg/ml Hoechst 33258 was used to stain nuclei. Images were taken under a spin‐disk confocal microscope (Andor Revolution, UK).

### 4.4 Chromosome spread

Acid Tyrode's solution (pH 2.5) was used to remove oocyte zona pellucida at 37°C for 30 seconds. Treated oocytes were transferred to MEM+ medium for 10 min. Permeating & fixing solution (1% PFA, 0.15% Triton X‐100 and 3 mM DTT) were loaded onto a glass slide, and oocytes were softly transferred into the solution. The slides were dried at room temperature and ready for immunostaining.

### 4.5 Immunoprecipitation

Note that, 2.5 μg of mouse anti‐flag, mouse anti‐strep II, rabbit anti‐Rad51, or rabbit anti‐p‐CHK2(Thr68) antibody was first incubated with 30 μl protein‐A/G beads (Yeasen Biotech Co., Shanghai, China) in 250 μl Lysis buffer (with protease inhibitor and phosphatase inhibitor, 1:500, Yeasen) on a rotator at 4°C for 4 h. Meanwhile, 2.5 × 10^6^ transfected 293T cells or two genital ridges were lysed and ultrasonicated in 250 μl IP buffer and then pre‐cleaned with 30 μl protein A/G beads at 4°C for 4 h. Then, the protein A/G‐coupled antibody was incubated with pre‐cleaned lysates at 4°C overnight. Next, after three washes with IP buffer, antibody‐bound beads were subjected to western blotting with corresponding antibodies.

### 4.6 In vivo ubiquitination assays

293T cells with 50%–70% confluence were transfected with FBXW24‐EGFP‐Strep II (WT, 3 M, 2 M, 5 M, K417, R421, or K435) and/or SYCP3‐TagRFP‐Flag (WT, 3 M, 1 M or 2 M) plasmids (in pcDNA3.1+) by Lipofectamine 3000 Transfection Reagent (Thermo Fisher). Note that, 48 h after transfection, cells were washed with PBS three times and dissolved in RIPA lysis buffer for 30 min on ice. Cells were collected and subjected to IP followed by western blot with anti‐Ubiquitin (A46) antibody. Plasmid construction information is in Table S3. The exact amount of plasmids used is described in related figure legends.

### 4.7 Purification of recombinant SYCP3 and FBXW24 proteins

We used the Bac‐to‐bac system (Thermo Fisher) to clone and express recombinant SYCP3 and FBXW24 proteins. Briefly, WT or mutant, *Fbxw24‐EGFP‐StrepII* or *Sycp3‐TagRFP‐Flag* DNA was cloned into pFastBacHTA (Thermo Fisher) and then transformed into DH10Bac *Escherichia coli* cells. We used QIAfilter Plasmid Purification Kit (Qiagen) to isolate bacmid from DH10Bac E. *coli* and then transfected the upper bacmids into Sf9 cells with Cellfectin® II Transfection reagent (Thermo Fisher). The first round of baculovirus titer was collected three days after. This process was repeated twice to amplify the titer. For protein expression, Sf9 cells in 250 ml SFM900‐II medium (Thermo Fisher) with 5% FBS were infected by 20 μl of the third titer for three days. Infected cells were dissolved with a high‐pressure cell disrupter (Union Biotech, Shanghai, China), centrifuged, and the resulting supernatant was incubated with 1 ml Ni‐NTA Superflow resin (Qiagen) at 4°C for 1 h, then eluted by 500 mM imidazole in resuspension buffer. The size‐exclusion spin column was used to concentrate eluted protein and exchanged into BRB80 (80 mM HEPES, 1 mM MgCl2, 1 mM EGTA, pH 6.8 by KOH) with 10% glycerol, 50 μM ATP, and 5 mM DTT. Buffer‐exchanged final protein stock was aliquoted and stored at –80°C. All Sf9 cultures are maintained in an incubator at 27°C.

### 4.8 In vitro ubiquitination assays

All commercial ubiquitination components were bought from R&D Systems Co. (Minneapolis, MN, USA). In vitro ubiquitination assays were discharged in a 15 μl ubiquitination reaction mixture including 10X reaction buffer, UBE1 (E1), UbcH5a/UBE2D1 (E2), CUL1/RBX1, SKP1/SKP2, Ubiquitin, purified SYCP3‐TagRFP or FBXW24‐EGFP protein (or both), ATP, and MgSO_4_. The reaction mixture was incubated at 30°C for 2 h. All information about the stock concentration (conc.), final conc, and final quantity are in Table S4. Lastly, the reaction was disposed of by western blot.

### 4.9 Quantification of immunofluorescence, live fluorescence and western blot images

For total intensity measurements, first, original tif images are opened in ImageJ (NIH), then signal intensity and background were measured; signal intensity minus background intensity will be the net intensity. For western blot or agarose gel, integrated intensity is obtained by multiplying the net intensity by the band area.

For SYCP3 intensity quantification in Figure S6C–E, first, the net SYCP3 or net centromere intensity was obtained by measured intensity subtracted by the background intensity. Then a ratio of SYCP3 net intensity/centromere net intensity was obtained to verify the increment of SYCP3 intensity in Fbxw24‐KO germ cells. For SYCP3 intensity quantification in all other figures, SYCP3 intensity is the measured intensity subtracted by the background intensity (net intensity).

For SYCP3 signal area delineation in the meiotic prophase, first, we open the original tif. image in Image J and draw a square around the chromosome region and run "duplicate" to show the chromosome region on a new board, then we run the "threshold" of "adjust" to delineate the chromosome region with SYCP3 signal; finally, we run "measure" of "analysis" to get the Sycp3 intensity value. For SYCP3 signal area delineation in MII oocytes (Figure 2A,B, Figure S6A,B), since the SYCP3 signal did not show a clear pattern, we have to delineate the DNA region as the region for SYCP3.

The chromosome foci of γH2AX, RPA2, RAD51, p‐CHK2, and MLH1 were manually counted by two independent authors, and the average number was used as the final number.

### 4.10 Animal/individual sample inclusion, experiment grouping, data collection, and data analysis

All selected mice (female) must be physically healthy, as judged by average weight, normal eating, daily activities, etc. Poor quality or unhealthy mice were excluded.

We tried to follow the blind roles in grouping all experiments, data collection, and analysis. Different authors perform the tasks of data collection, data analysis, and data inputting (into excel files).

For experiment grouping and data collection in fertility assays (Figure 1L), each mating cage was assigned a different cage number but was not labelled "WT" or "FBXW24‐KO". Each cage is checked daily by an author (who recorded newborns), and the data were handed to the first author. The first author entered daily data into an excel file, in which each cage number corresponded to a specific WT or *Fbxw24*‐KO female mice.

All other experimental groups and data collection, control, or *Fbxw24*‐KO samples must be marked. However, during image collection, follicular counting, or intensity quantification, to eliminate the subjectivity of the data collector, the group labels of each sample are covered with black sticky wires and relabeled as numbers or letters. After processing, the black sticky thread was removed, the correlation between the analysis data and the sample information was determined by the first author, and the data was input into the corresponding original excel file by the first author.

Before the experiment, each individual (oocyte, ovary, or mouse) was selected in an independent replicate or group and randomly assigned blindly. For independently repeated data collection, each data point was randomly selected.

### 4.11 Statistical analysis

All experiments were repeated at least three times. The data are expressed as mean ± SEM. The comparison between the two groups was carried out by the Student's *t*‐test. One‐way ANOVA (analysis of variance) was used to compare the differences between three or more groups, and the *p*‐value < 0.05 was statistically significant. Independent repeat times for western blot are always 3 (unless stated otherwise) and are no longer labelled in the related quantification graph. For all other quantification graphs, independent repeat times (*R* = ) and/or independent sample numbers used for measurement (*n* = ) were labelled in the graph. GraphPad Prism 9 was used for statistical analysis.

## CONFLICT OF INTEREST

The authors declare no conflict of interest.

## FUNDING INFORMATION

This work was financially supported by the National Key Research and Development Program of China (Grant No: 2018YFC1003400 to Dong Zhang; Grant No: 2018YFA0107701 to Qing‐Yuan Sun; Grant No: 2018YFC1004203 to Jing Li), the National Natural Science Foundation of China (Grant No: 32070840) to Dong Zhang, and the Innovative Project of Scientific Research Program of Jiangsu Province to Wen‐Yi Gao (Grant No: JX11613622).

## Supporting information

Supporting Information

Supporting Information

Supporting Information

Supporting Information

Supporting Information

Supporting Information

Supporting Information

Supporting Information

Supporting Information

Supporting Information

Supporting Information

Supporting Information

Supporting Information

Supporting Information

Supporting Information

Supporting Information

Supporting Information

Supporting Information

Supporting Information

Supporting Information

Supporting Information

Supporting Information

Supporting Information

Supporting Information

Supporting Information
