# Supplementary material for: FBXW24 controls female meiotic prophase progression by regulating SYCP3 ubiquitination
Source: Clin Transl Med. 2022 Jul 20;12(7):e891. doi: 10.1002/ctm2.891 (PMC9299759; doi:10.1002/ctm2.891)
Supplement: Supplementary file 1 — Supporting Information [file CTM2-12-e891-s002.doc]

**FBXW24 controls female meiotic prophase progression by regulating SYCP3 ubiquitination**

Yang Wang^1, 6^, Wen-Yi Gao^1, 6^, Li-Li Wang^1, 6^, Ruo-Lei Wang^1, 6^, Zhi-Xia Yang^1, 6^, Fu-Qiang Luo^1^, Yu-Hao He^1^, Zi-Bin Wang^3^, Fu-Qiang Wang^3^, Qing-Yuan Sun^2, 5^, Jing Li^1, 5^ and Dong Zhang^1, 4, 5^

^1^ State Key Lab of Reproductive Medicine, Nanjing Medical University, Nanjing, 211166, Jiangsu, China

^2^ Fertility Preservation Lab and Guangdong-Hong Kong Metabolism & Reproduction Joint Laboratory, Reproductive Medicine Center, Guangdong Second Provincial General Hospital, Guangzhou, 510317, Guangdong, China

^3^ Analysis and Test Center, Nanjing Medical University, 101 Longmian Ave. Nanjing, 211166, Jiangsu, China

^4^ Animal Core Facility, Nanjing Medical University, Nanjing, 211166, Jiangsu, P.R.China

^5^ To whom correspondence should be addressed to:

**Dong Zhang:** State Key Lab of Reproductive Medicine, Nanjing Medical University, 101 Longmian Ave., Nanjing, 211166, Jiangsu, China; dong.ray.zhang@njmu.edu.cn; Tel. +86-25-86869504; Fax. +86-25-86869504.

**Jing Li:** State Key Lab of Reproductive Medicine, Nanjing Medical University, 101 Longmian Ave., Nanjing, 211166, Jiangsu, China; ljwth@njmu.edu.cn; Tel. +86-25-86869502; Fax. +86-25-86869502.

**Qing-Yuan Sun:** Fertility Preservation Lab and Guangdong-Hong Kong Metabolism & Reproduction Joint Laboratory, Reproductive Medicine Center, Guangdong Second Provincial General Hospital, 466 Xingangzhong Road, Guangzhou, 510317, Guangdong, China; sunqy@gd2h.org.cn; Tel. +86-10-64807050; Fax. +86-10-64807050

^6^ Equal contribution

**Running title**

FBXW24 Ubiquitinates SYCP3 during Meiotic Prophase

**Supplementary MATERRIALS AND METHODS**

**Oocyte collection and in vitro culture**

Fully-grown GV oocytes were collected from three-week-old female mice. Oocytes were released by puncturing follicles with a sterile syringe needle in MEM+ medium (0.01 mM EDTA, 0.23 mM Na-pyruvate, 0.2 mM penicillin/streptomycin and 3 mg/ml BSA in MEM). After washing away the cumulus cells from the cumulus-oocyte complexes, oocytes were cultured in 100µl mini-drops of MEM+ containing 20% fetal bovine serum (FBS) (Thermo Fisher) covered with mineral oil at 37.0 °C in an incubator with 5% O_2_, 5% CO_2_, and humidified atmosphere.

**Cell culture and plasmid transfection**

Human embryonic kidney (HEK-293FT) cells were cultured in DMEM with 10% FBS. For plasmid transfections, cells were grown at 50-70% confluence, and were transfected with Lipofectamine^TM^ 3000 Transfection Reagent (Thermo Fisher) according to the manufacturer’s protocol.

**SiRNA production and RNA interference**

To verified the specificity of SYCP3, RAD51, p-CHK2, γH2AX, STAT1 and DTX3L (Supp. Fig. 17), siRNA were produced and purified by using the T7 Ribomax Express RNAi System (Promega, Madison, WI, USA) according to the manufacturer’s instructions. Purified siRNAs were aliquoted and stored at -80°C after a quality check on an agarose gel. A ready-to-use siRNA mixture was set by mixing siRNAs against four distinct target regions at an equal molar ratio to a final concentration of 5 µM. DNA templates for siRNAs were selected also according to the manufacturer’s instructions. All template sequences are in Supp. Table 6.

For SYCP3 knockdown, we cultured five 18.5 dpc genital ridge on the surface of a Millicell cell culture insert (0.4 µm in pore size, 12 mm in diameter and 10.5 mm in height, Merck, Darmstadt, Germany) in one well of 24-well plate with 0.5 ml MEM-α (Thermo) with 10% FBS (Thermo). siRNA were transfected with Lipo8000 (Beyotime, Beijing) according the reagent's manual. Sample were collected 48 hour afterwards.

For knockdown of other proteins, we plated MCA205 cells into 24-well plate at 40% confluence. siRNA were also transfected with Lipo8000. Sample were collected 48 hour afterwards.

**Immunofluorescence staining of oocytes**

Oocytes were permeabilized with 0.5% Triton X-100/PHEM (60 mM PIPES, 25 mM Hepes, pH 6.9, 10 mM EGTA, 8 mM MgSO_4_) for 5 minutes, and then fixed in 3.7% FPA in PHEM for 20 minutes at room temperature. After being washed with PBS/0.05% PVP (polyvinylpyrrolidone) for 10 min three times, oocytes were blocked in blocking buffer (100 mM glycine and 1% BSA in PBS) for 1 hour at room temperature. Then primary antibody was diluted in blocking buffer and oocytes were incubated in it overnight at 4.0 °C. The subsequent steps were performed as described above. Secondary antibodies: horseradish peroxidase (HRP)-conjugated rabbit anti-goat IgG and HRP-conjugated goat anti-mouse IgG were purchased from Vazyme (Nanjing, Jiangsu, China). Cy2-conjugated donkey anti-mouse IgG (Code: 715-225-150), Rhodamine (TRITC)-conjugated donkey anti-human IgG (Code: 709-025-149), Cy2-conjugated donkey anti-human IgG (Code: 709-225-149), and Cy2-conjugated donkey anti-rabbit IgG (Code: 711-225-152), were purchased from Jackson ImmunoResearch Laboratory (West Grove, PA, USA).

**RNA isolation and real time-qPCR**

Total RNA was extracted from tissues and oocytes using a RNAprep pure Tissue (TIANGEN Biotech, Beijing, China) according to the manufacturer’s protocol. RNA was reverse transcribed into cDNA with a FastQuant RT Kit (TIANGEN Biotech) according to the manufacturer’s protocol. Real time-quantitative PCR was then performed with Eva Green qPCR Master Mix using the following cycling conditions: Initial denaturation at 95 ˚C for 5 min, followed by 40 cycles at 95 ˚C for 5 sec and at 60 ˚C for 30 sec. *β-Actin* was used as an internal control for normalization. The specific primers used in the experiment are shown in supplementary table 1 and 2. Experiments were all repeated in triplicate.

**Histological immunofluorescence analysis and ovary follicle counting**

Ovaries were obtained, washed and fixed in 10% buffered formalin or 4% PFA overnight, embedded in paraffin, continuously sectioned at 5 µm thickness, then stained with hematoxylin and eosin. The follicle stages were classified according to Pederson's standard. Only follicles with a visible nucleus were counted. Briefly, an oocyte surrounded by a single layer of flattened or cubical granulosa cells was defined as a primordial or primary follicle; an oocyte surrounded by more than one layer of cuboidal granulosa cells without visible antrum was defined as a secondary follicle; a follicle clearly having an antral space containing follicular fluid was defined as antral follicle. The number of follicles counted per ovary was used for statistical analysis.

**Coomassie staining and characterization of SYCP3 ubiquitination sites**

For coomassie staining, SYCP3 were separated on an SDS-PAGE gel and stained in a coomassie staining solution (0.1% Coomassie® R-250 in 40% ethanol, 10% acetic acid) for 30 minutes at room temperature and then destained in a destaining solution (10% ethanol and 7.5% acetic acid) for several hours, then several bands above the expected-size band that are the presumabl ubiquitinated portion were cut out and sent to Hangzhou Jingjie Co. (Hangzhou, Zhejiang, China) for LC-MS characterization of ubiquitination sites.

**Yeast two-hybrid assays**

The coding sequence of *Sycp3* was cloned into pGADT7, and full-length *Fbxw24*, *Fbxw24* *1-4*, or *Rad51* were cloned into pGBKT7. Two plasmids were together transformed into yeast strain AH109. Empty vectors were used as negative controls. The transformed yeast was grown on synthetically defined SD/-Trp-Leu dropout medium plates for 2-4 days. The yeast cells with co-transformation of the two fusion plasmids were further dropped on SD/-Trp-Leu-His-Ade dropout medium plates for further verification of the interactions.

**Immunogold-electron microscopy**

Digested single cells from 16.5 DPC genital ridge were mounted onto a slide and blocked with 1% BSA, incubated with SYCP3 Ab (4 °C, overnight) and then labelled with Donkey anti-rabbit IgG/Gold (35 nm) at room temperature for 2 hours. Then, oocytes were re-blocked with 1% BSA (to eliminate the cross reaction between two antibodies), incubated with FBXW24 Ab (4 °C, overnight) and then labelled with Donkey anti-rabbit IgG/Gold (15 nm) at room temperature for 2 hours.

After being labelled, cells were fixed in 2.5% glutaraldehyde for 2 hours at 4 °C, washed three times with PBS and stained with eosin for 2 minutes to facilitate the oocyte positioning by eye. Oocytes were then placed in 2% agarose and spun for 5 minutes at 13000 rpm and held overnight at 4 °C. The next day, the agarose piece with eosin-stained oocytes (red in the piece) was trimmed and sent to the Testing and Analysis Center (Nanjing Medical University) for sample preparation for transmission electron microscopy. Electron microscopy pictures were obtained with a transmission electron microscope (FEI Tecnai G2 Spirit Bio Twin; Thermo Fisher Scientific).

**TMT-labeling quantitative proteomics**

About fifty three-week ovaries per sample (about 50 mg), two repeats for WT and *Fbxw24*-KO, respectively, were sent to Hangzhou Jingjie Co. (Hangzhou, China). Briefly, ovaries were cracked and the supernatant was digested by trypsin into peptides. Then the peptides from individual samples was isobaric-mass tagged by TMT6-126, 127, 128, and 129, respectively, according to the manufacturer's protocol for TMT kit/iTRAQ kit (Thermo Fisher). Next, TMT-labeled tryptic peptides were fractionated into fractions by high pH reverse-phase HPLC using Thermo Betasil C18 column, then peptide fractions were subjected to NSI source followed by tandem mass spectrometry (MS/MS) in Q ExactiveTM Plus (Thermo) coupled online to the UPLC. The resulting MS/MS data were processed using Maxquant search engine (v.1.5.2.8).

**Supplementary Figure Legends**

**Supplementary Figure 1. Expression and localization of FBXW24 within oocytes.**

a. Western blot in oocytes shows that upon meiosis resumption (GVBD), FBXW24 sharply decreased. b. Immunofluorescence shows that at GV stage, FBXW24 is relatively rich within the nucleus and distributed within the cytoplasm and cortex; at GVBD or MI stage, FBXW24 sharply diminishes. c. Immunofluorescence shows that once the microtubule cytoskeleton was disassembled by nocodazole (Noc), FBXW24 did not demonstrate any enrichment within the nucleus. d. Immunofluorescence shows that once the actin cytoskeleton was disassembled by Cytochalasin D (Cyto-D), FBXW24 did not show any enrichment within the nucleus. GAPDH was used as loading control. Scale bar, 20 µm.

**Supplementary Figure 2. *Fbxw24* knockout impede follicle development and meiotic defect within oocytes.**

a and B. At PND 3, *Fbxw24* knockout significantly increased primordial follicles (PMF) while decreased primary follicles (PF). Four selected regions (red dot-line square) from WT and KO ovaries were zoomed and placed on the right. C. Immunofluorescence shows that *Fbxw24*-KO oocytes have decreased spindle microtubules, misaligned chromosomes, and altered geometry. D–F. Quantification of MI spindle intensity (D), spindle length (E), and spindle width (F). Scale bar in A, 100 µm; scale bar in C, 20 µm. **, p < 0.01; ****, p < 0.0001.

**Supplementary Figure 3. *Fbxw24* knockout increased RPA2 foci in GV oocytes.**

a and B. Immunofluorescence and quantification demonstrates that *Fbxw24* knockout significantly increased RPA2 in GV oocytes. DNA in blue, RPA2 in green. Scale bar, 20 µm; **, p < 0.01.

**Supplementary Figure 4. *Fbxw24* knockout decreased autophagy in oocytes.**

a and B. Immunofluorescence and quantification demonstrates that *Fbxw24* knockout significantly increased LC3B in GV oocytes. DNA in blue, LC3B in green. C and D. Western blot and quantification shows that *Fbxw24* knockout significantly increased LC3B level in GV oocytes. "Int." in the Y-axis is an abbreviation of "intensity". Scale bar, 20 µm. Actin was used as loading control. *, p < 0.05; ****, p < 0.0001.

**Supplementary Figure 5. *Fbxw24* knockout altered chromatin status and maternal mRNA transcription**

A and B. *Fbxw24* knockout significantly decreased the percentage of GVBD in mouse oocytes. A and C. *Fbxw24* knockout significantly decreased the oocyte diameter (abbreviated as "Dia." in the Y-axis). D and E. *Fbxw24* knockout significantly decreased the percentage (abbreviated as "Pct." in the Y-axis) of oocytes with mature nuclear configuration (SN, surrounded nucleus). F. Western blot shows that *Fbxw24*-KO oocytes had elevated H2FY level, while H3K4 and H3K27 levels were maintained. G. Q-PCR shows that *Fbxw24*-KO oocytes had dramatically elevated levels of maternal mRNAs, including *Gdf9, Bmp15, Zp3*, and *Nobox.* GAPDH was used as loading control. Scale bar in A, 100 μm; scale bar in D, 20 μm. **, p < 0.01; ***, p < 0.001; ****, p < 0.0001.

**Supplementary Figure 6. Further verification of the SYCP3 increment in *Fbxw24*-KO female germ cells**

a and B. Immunostaining with another rabbit anti-SYCP3 antibody (Cat#: ab15093; Abcam) on spreaded chromosomes and quantification demonstrates that *Fbxw24* knockout significantly increased SYCP3 intensity within pachytene germ cells and MII oocytes. Moreover, Fbxw24 knockout significantly lessened the reduction of SYCP3 intensity from pachytene to MII. DNA in blue, SYCP3 in red. C-G. immunostaining and quantification showed that there is no difference for centromere intensity between WT and *Fbxw24-*KO germ cells, meanwhile SYCP3 intensity significantly increased in *Fbxw24-*KO germ cells. DNA in blue, SYCP3 in red, centromere in green. H and I. immunostaining and quantification showed that *Fbxw24* knockout significantly increased SYCP3 intensity within diplotene germ cells. DNA in blue, SYCP3 in red. Scale bars, 20 µm. ***, p < 0.001; ****, p < 0.0001.

**Supplementary Figure 7. *Fbxw24* knockout didn't affect synapsis**

a. Immunostaining demonstrates that *Fbxw24* knockout didn't affect synapsis at all. DNA in blue, SYCP1 in green, SYCP3 in red. Scale bar, 100 µm.

**Supplementary Figure 8. Yeast two-hybridization verification of the direct interaction between FBXW24 and SYCP3**

a. Yeast two-hybridization showed that JM109 strain transfected with both pGBK-*Fbxw24* and pGAD-*Sycp3* grew well on both -Trp-Leu and -Trp-Leu-His-Ade solid medium, suggesting that FBXW24 directly bind SYCP3. Empty plate, pGBK + pGAD empty vector, pGBK-*Fbxw24* + pGAD and pGBK + pGAD-*Sycp3* were used as controls.

**Supplementary Figure 9. Side-by-side comparison of in-vitro ubiquitination reaction in the absence of individual components**

a. Western blot showed that, in in-vitro ubiquitination reaction, if all components were added, there are obvious ubiquitination level. if any of the components was removed, there was very low ubiquitination level. Ubiquitinated SYCP3 was detected by Ub46 antibody. B. Over-exposure of the same membrane in A. C. The experimental setting is the same as in A, and ubiquitinated SYCP3 was detected by SYCP3 antibody. If all components were added, SYCP3 antibody detects much wider range of bands, while in all other reactions without some component, SYCP3 antibody detects much narrower range of bands. 1μg SYCP3-Flag and/or FBXW24-StrepII were/was used in each reaction.

**Supplementary Figure 10. In-vivo dose-dependent degradation of SYCP3 by FBXW24**

a. In-vivo FBXW24 dose-dependent SYCP3 degradation in sf9 cells showed that as FBXW24 increased, SYCP3 intensity gradually reduced. FBXW24-EGFP in green, SYCP3-TagRFP in red. B. Quantification of A. Scale bar, 20 µm.

**Supplementary Figure 11. Spectrum map for identification of Sycp3 ubiquitination sites.**

a-i. Spectrum map for identification of Sycp3 ubiquitin sites. These sites are also in Supp. Table 5. From a to i, K34, K104, K119, K124, K130, K177, K223, and K232.

**Supplementary Figure 12. Structure model of FBXW24-WT and mutant of each single site in FBXW24-4.**

a. Structure model of FBXW24-WT created by I-Tasser. b–I. Structure model of FBXW24-R304A, R305A, R307A, K417A, R421A, K354A, R387A, and K435A. The relative N- and C-terminal orientations for all models were aligned in the same manner.

**Supplementary Figure 13. Stability comparison between WT and FBXW24-2M, 3M, and 5M**

a-C. Same amount of FBXW24-WT, 2M, 3M or 5M titer was added into sf9 medium and FBXW24 was expressed for 1, 2 and 3 days. Western blot and quantification showed that the protein stability was similar between FBXW24-WT, 2M, 3M or 5M. D. Co-IP and western blot showed that the interaction between SYCP3-Flag and any FBXW24-StrepII protein (WT, 2M, 3M or 5M) were similar.

**Supplementary Figure 14. Stability comparison between WT and FBXW24-****K354A, R387A, and K435A**

a-C. Same amount of FBXW24-WT, K354A, R387A, and K435A titer was added into sf9 medium and FBXW24 was expressed for 1, 2 and 3 days. Western blot and quantification showed that the protein stability was similar between FBXW24-WT, K354A, R387A, and K435A. D. Co-IP and western blot showed that the interaction between SYCP3-Flag and any FBXW24-StrepII protein (WT, K354A, R387A, and K435A) were similar.

**Supplementary Figure 15. Protein sequence alignment between mouse FBXW24 and human FBXW7-iso1**

Protein sequence alignment between mouse FBXW24 and human FBXW7-iso1 showed that the similarity of WD domain between mouse FBXW24 (84-486 AA) and human FBXW7 is pretty low.

**Supplementary Figure 16.** ***Fbxw24* knockout did not alter the level of Hormad1 and Trip13.**

a and b. Western blot and quantification showed that Hormad1 level did not alter in the 16.5 DPC *Fbxw24*-KO genital ridge. C and d. Western blot and quantification shows that Trip13 level did not alter in the 16.5 DPC *Fbxw24*-KO genital ridge. Actin was used as loading control.

**Supplementary Figure 17. Verification of antibody specificity.**

a-L. Western blot and quantification showed that siRNA knockdown significantly reduced the protein level, as detected by corresponding antibody, suggesting that the corresponding antibody is fairly specific.

**Supplementary dataset Legends**

**Supplementary Dataset 1.**

Related to quantitative proteomics in Fig. 8D-G. This excel file contains two sheets. ">1.2 fold upregulated" or "< 0.833 fold downregulated" included all of upregulated (*Fbxw24*-KO / WT > 1.2) or downregulated (*Fbxw24*-KO / WT < 0.833) DEPs (differentially-expressed proteins).

**Supplementary Dataset 2.**

Related to Fig. 5A and results part (page 9, line 19-22). This excel file contains two sheets. "All proteins" contained information for all proteins that the ubiquitinated peptides corresponded to; "SYCP3 ubiquitinated sites" included all identified SYCP3 sites (highlighted in yellow or blue) from immunocomplex with SYCP3 IP in 18.5 dpc genital ridges. Among these highlighted sites. These five sites that overlaped with the identified ubiquitination sites from sf9 cell-purified SYCP3-Flag protein were highlighted in blue.

**Supplementary Movie Legends**

**Supplementary movie 1. *Fbxw24*-KO oocyte was unable to progress into MII due to failure in homologous chromosome segregation.**

Related to Fig. 1S. Time-lapse movie of meiosis in WT or *Fbxw24*-KO oocytes shows that after 14 h of IVM, the chromosomes in WT oocyte have separated in a timely manner, and the first polar body extruded; in contrast, the chromosomes in *Fbxw24*-KO oocyte cannot align well at the metaphase plate and go through anaphase I into MII. The movie was taken 3 h after IVM (which corresponded to early pro-metaphase) at a time interval of 30 min.

**Supplementary Movie 2. Time lapse comparison of the increment extent of SYCP3 intensity with or without FBXW24**

Related to Fig. 4I and 4J. We first added Sycp3 titer to expressed to a medium level (at about Day 1.5), then added FBXW24 titer, we saw the Sycp3 intensity increased fairly slow, in contrast, in the group without FBXW24 supplement, Sycp3 intensity keep raising and big Sycp3 aggreagtes kept increasing. FBXW24-Strep II in green, SYCP3-Flag in red. time interval, 30 min.

**Supplementary Movie 3. Six selected ubiquitination sites for mutant study in Sycp3.**

Related to Figure 5c. Cartoon movie of Sycp3 tetramer. Selected ubiquitination sites are highlighted in red. The tetramer is rotated 360^o^ first along X-axis and then along Y-axis.

**Supplementary Movie 4. Five selected lysine (K) or arginine (R) sites for mutant study in FBXW24.**

Related to Figure 6a and 6c–e. Cartoon movie of FBXW24 model. FBXW24-1 is in dark blue; FBXW24-2 is in light blue; FBXW24-3 is in pink; and FBXW24-4 is in green. Six selected lysine (K) or arginine (R) sites are highlighted in red. The model is rotated 360^o^ first along X-axis and then along Y-axis.

**Supplementary Movie 5. Side-by-side comparison of the WD40 domain between mouse FBXW24 and human FBXW7.**

Structure model of FBXW24 WD40 domain (right model) and FBXW7 WD40 domain (left model) were highly similar. FBXW24-R304A, R305A, R307A, K417A, and R421A were red-highlighted, K354A, R387A, and K435A were blue-highlighted.

**Supplementary Tables**

**Supplementary table 1. RT-PCR primers for mouse *Fbxw*** family members

| **Name** | | **Sequence** | **Length** |
| --- | --- | --- | --- |
| ***Fbxw2*** | **Forward** | 5'-GATGGACTTCTCTGTACAGGGTC | 296bp |
|  | **Reverse** | 5'-GATAAAGCCCATACTTTCACAGC |  |
| ***Fbxw4*** | **Forward** | 5'-CGTGAAACTGTCTCAGAACTGG | 291bp |
|  | **Reverse** | 5'-GTGCTGTGGATCTTGTGAACAC |  |
| ***Fbxw5*** | **Forward** | 5'-GATGACTCACTGCTGCTGG | 296bp |
|  | **Reverse** | 5'-CTGGCATTGAGGTTCTGG |  |
| ***Fbxw7*** | **Forward** | 5'-GGAACAGGAGGAGGAAGAGG | 306bp |
|  | **Reverse** | 5'-GTTGCTGAACATGGTACAAGG |  |
| ***Fbxw8*** | **Forward** | 5'-ACTTGAATGAGCTGGATGACG | 308bp |
|  | **Reverse** | 5'-ATGCTCCAGCTCGCTCAC |  |
| ***Fbxw9*** | **Forward** | 5'-GCTGCTGGAGATCTGTGC | 295bp |
|  | **Reverse** | 5'-ACCCTGGAGCAGCAACAC |  |
| ***Fbxw10*** | **Forward** | 5'-GAAGATGAAGGAGATCCTCTACTGG | 276bp |
|  | **Reverse** | 5'-GACATTGGGAATGACACAGG |  |
| ***Fbxw11*** | **Forward** | 5'-TGCCAGGTCTCTGTGTGC | 279bp |
|  | **Reverse** | 5'-ATCCTCTGCAAGTTGTGTCG |  |
| ***Fbxw13*** | **Forward** | 5'-AGGCCAGTCGTCTGTATGG | 294bp |
|  | **Reverse** | 5'-GAGGTTTCCGGAGATATCACC |  |
| ***Fbxw14*** | **Forward** | 5'-ATGAGGGGACACCAGACC | 278bp |
|  | **Reverse** | 5'-AATGTAGCCAGACATTTGCTG |  |
| ***Fbxw15*** | **Forward** | 5'-GATTCCAGATTTATACCTCATTTCC | 316bp |
|  | **Reverse** | 5'-CCCAAACTCTTCCAGCTTG |  |
| ***Fbxw16*** | **Forward** | 5'-TCTCCCTGTCAAATACTGAAAGC | 304bp |
|  | **Reverse** | 5'-AGAAGACTCCTTGGCATAATGG |  |
| ***Fbxw17*** | **Forward** | 5'-ACAGGATTTCATCTGCAAAGC | 303bp |
|  | **Reverse** | 5'-AAGAAGGCCCACTCAGACC |  |
| ***Fbxw18*** | **Forward** | 5'-CCGAACATATCAAGAACACACC | 298bp |
|  | **Reverse** | 5'-AGTTGAGTGAATATCTACAGTGACTGC |  |
| ***Fbxw19*** | **Forward** | 5'-CAAAGACATGTCTTGACAAAGGTG | 275bp |
|  | **Reverse** | 5'-CGCTCCTTGCAGTCTTGC |  |
| ***Fbxw20*** | **Forward** | 5'-GGTCATCTGTATGGTGAGTTCC | 283bp |
|  | **Reverse** | 5'-TCCCGAGATATCGCCTACC |  |
| ***Fbxw21*** | **Forward** | 5'-CTCCTGGGTAAAGAGACATGG | 304bp |
|  | **Reverse** | 5'-ACATCTCAGGGAGGGTAGTCAAC |  |
| ***Fbxw22*** | **Forward** | 5'-GGTGACTTCCGTGAACAGG | 270bp |
|  | **Reverse** | 5'-CCACAGACATCTCCTACCAAGAC |  |
| ***Fbxw23*** | **Forward** | 5'-TGTGGAGTTTGGTATAAGCTTGG | 292bp |
|  | **Reverse** | 5'-CTTAGTTGCCAGAGCATCACTG |  |
| ***Fbxw24*** | **Forward** | 5'-CAGGTTACATATCAGGCTGTGC | 301bp |
|  | **Reverse** | 5'-CCATCCTTGGAAATGACAGC |  |
| ***Fbxw26*** | **Forward** | 5'-AATGGACAAGGGAAGTCTGC | 306bp |
|  | **Reverse** | 5'-CATTTCTGGACATCAAGGTTATCC |  |
| ***Fbxw27*** | **Forward** | 5'-TTTGCAATATGGACCTTGTGG | 302bp |
|  | **Reverse** | 5'-GTGATTTGCATGGAGATTCC |  |
| ***Fbxw28*** | **Forward** | 5'-ACAAGAAAAGTCTGCTGTCTGTATG | 275bp |
|  | **Reverse** | 5'-CCACAGGCATCACCTACC |  |

**Supplementary table 2. Q-PCR primers of maternal factors**

| **Name** | | **Sequence** |
| --- | --- | --- |
| ***Gdf9*** | **Forward** | 5'-GGCATATGGGTGTACAGGGG |
|  | **Reverse** | 5'-ACGCAGTAGGCACACATCAT |
| ***Bmp15*** | **Forward** | 5'-TCCTTGCTGACGACCCTACAT |
|  | **Reverse** | 5'-TACCTCAGGGGATAGCCTTGG |
| ***Zp3*** | **Forward** | 5'-ATGGCGTCAAGCTATTTCCTC |
|  | **Reverse** | 5'-CGTGCCAAAAAGGTCTCTACT |
| ***Setd1b*** | **Forward** | 5'-TCCTCAAGCTCCGACAAGGAT |
|  | **Reverse** | 5'-CGTCGATGTCTGAATCAATCTGG |
| ***Nobox*** | **Forward** | 5'-AAGACCCGAACCCTGTACC |
|  | **Reverse** | 5'-CTCATGGCGTTTGTCACTGTC |
| ***Sycp1*** | **Forward** | 5'-TGAGGGGAAGCTCACGGTT |
|  | **Reverse** | 5'-CGAACAGTGTGAAGGGCTTTTG |
| ***Sycp3*** | **Forward** | 5'-AGCCAGTAACCAGAAAATTGAGC |
|  | **Reverse** | 5'-CCACTGCTGCAACACATTCATA |
| ***Chk2*** | **Forward** | 5'-GACAGTGCTTCCTGTTCACA |
|  | **Reverse** | 5'-GAGCTGGACGAACCCTGATA |
| ***Actin*** | **Forward** | 5'-CCGTAAAGACCTCTATGCC |
|  | **Reverse** | 5'-CTCAGTAACAGTCCGCCTA |

**Supplementary table 3. Plasmid construction primers for mouse *Fbxw24* and *Sycp3***

| **Name** | **Sequence** | **Length** |
| --- | --- | --- |
| ***Fbxw24*-SalI-F in pFastBac-strepII-EGFP** | 5'-ACGCGTCGACTAGAGATCCATTTGTCTAGTTTCCCTATG | 1401bp |
| ***Fbxw24*-NotI-R in pFastBac-strepII-EGFP** | 5'-AAGGAAAAAAGCGGCCGCAGAGCAGATGTTCAAGGTATATGC |
| ***Sycp3*-SalI-F in**  **pFastbac-flag-TagRFP** | 5'-ACGCGTCGACTACTTCGAGGGTGTGGGGACAG | 765bp |
| ***Sycp3*-NotI-R in**  **pFastbac-flag-TagRFP** | 5'-AAGGAAAAAAGCGGCCGCGAATAACATGGATTGAAGAGACTTTCG |
| ***Fbxw24-*NheI-F-in pcDNA -strepII-EGFP** | 5'-CTAGCTAGCGCCACCATGGAGATCCATTTGTCTAGTTTCCCTATG | 1401bp |
| ***Fbxw24-*HindIII-R-in pcDNA -strepII-EGFP** | 5'-CCCAAGCTTTTACTTCTCGAACTGTGGATGACTC |
| ***Sycp3*-EcoRI-F-in**  **pcDNA3.1-flag-TagRFP** | 5'-CCGGAATTCATGCTTCGAGGGTGTGGGGACAG | 765bp |
| ***Sycp3*-XhoI-R-in**  **pcDNA3.1-flag-TagRFP** | 5'-CCGCTCGAGCTAGAATAACATGGATTGAAGAGACTTTCG |
| ***Fbxw24*-NocI-F in pGBDT7** | 5'-CATGCCATGGTAGAGATCCATTTGTCTAGTTTCCCTATG | 1401bp |
| ***Fbxw24*-SalI-R in pGBDT7** | 5'-ACGCGTCGACTAGAGCAGATGTTCAAGGTATATGC |
| ***Sycp3*-EcoRI -F in GADT7** | 5'-CCGGAATTCCTTCGAGGGTGTGGGGACAG | 765bp |
| ***Sycp3*-BamHI-R in pGADT7** | 5'-CGCGGATCCAGAATAACATGGATTGAAGAGACTTTCG |
| ***Fbxw24*-1-NocI-F in pGBDT7** | 5'-CATGCCATGGTAGAGATCCATTTGTCTAGTTTCCCTATG | 312bp |
| ***Fbxw24*-1- EcoRI-R in pGBDT7** | 5'-CCGGAATTCACCTGCATATCCCCGAACAC |
| ***Fbxw24*-2-EcoRI -F in pGBDT7** | 5'-CCGGAATTCGGTGTTCGGGGATATGCAG | 294bp |
| ***Fbxw24*-2-BamHI -R in pGBDT7** | 5'-CGCGGATCCACAGTGATTTACATGGAGATTCCAG |
| ***Fbxw24*-3-BamHI-F in pGBDT7** | 5'-CGCGGATCCATCTGGAATCTCCATGTAAATCACTG | 387bp |
| ***Fbxw24*-3-SalI-R in pGBDT7** | 5'-ACGCGTCGACTAAATGTTTCAAACCTTGTGACTATGG |
| ***Fbxw24*-4-EcoRI -F in pGBDT7** | 5'-CCGGAATTCCCCATAGTCACAAGGTTTGAAAC | 480bp |
| ***Fbxw24*-4-SalI-R in pGBDT7** | 5'-ACGCGTCGACTAGAGCAGATGTTCAAGGTATATGC |
| ***Rad51*- BamHI -F in pGADT7** | 5'-CGCGGATCCTAGCTATGCAAATGCAGCTTGAAGC | 1014bp |
| ***Rad51*-XohI-R in pGADT7** | 5'-CCGCTCGAGTGTCTTTGGCATCGCCCACTC |

**Supplementary t**able 4. In-vitro ubiquitination reaction components

| **Components** | **Stock conc. (μM)** | **Final conc. (μM)** | **Volume (μl)** |
| --- | --- | --- | --- |
| 10 x reaction buffer | 10 x | 1 x | 1.5 |
| UBE1 (E1) | 5 | 0.2 | 0.6 |
| UbcH5a/UBE2D1 (E2) | 25 | 1 | 0.6 |
| Cul1/Rbx1 | 23 | 0.25 | 0.16 |
| Skp1/2 | 15 | 0.25 | 0.25 |
| Ubiquitin | 30 (μg/μl) | 30 (μg/μl) | 0.25 |
| Sycp3-TagRFP | 20 | 0.6 | 0.3 |
| FBXW24-EGFP | 20 | 0.6 | 0.3 |
| ATP | 75 | 1 | 0.2 |
| MgSO_4_ | 50 | 1 | 0.3 |
| ddH_2_0 |  |  | to 15 µl |


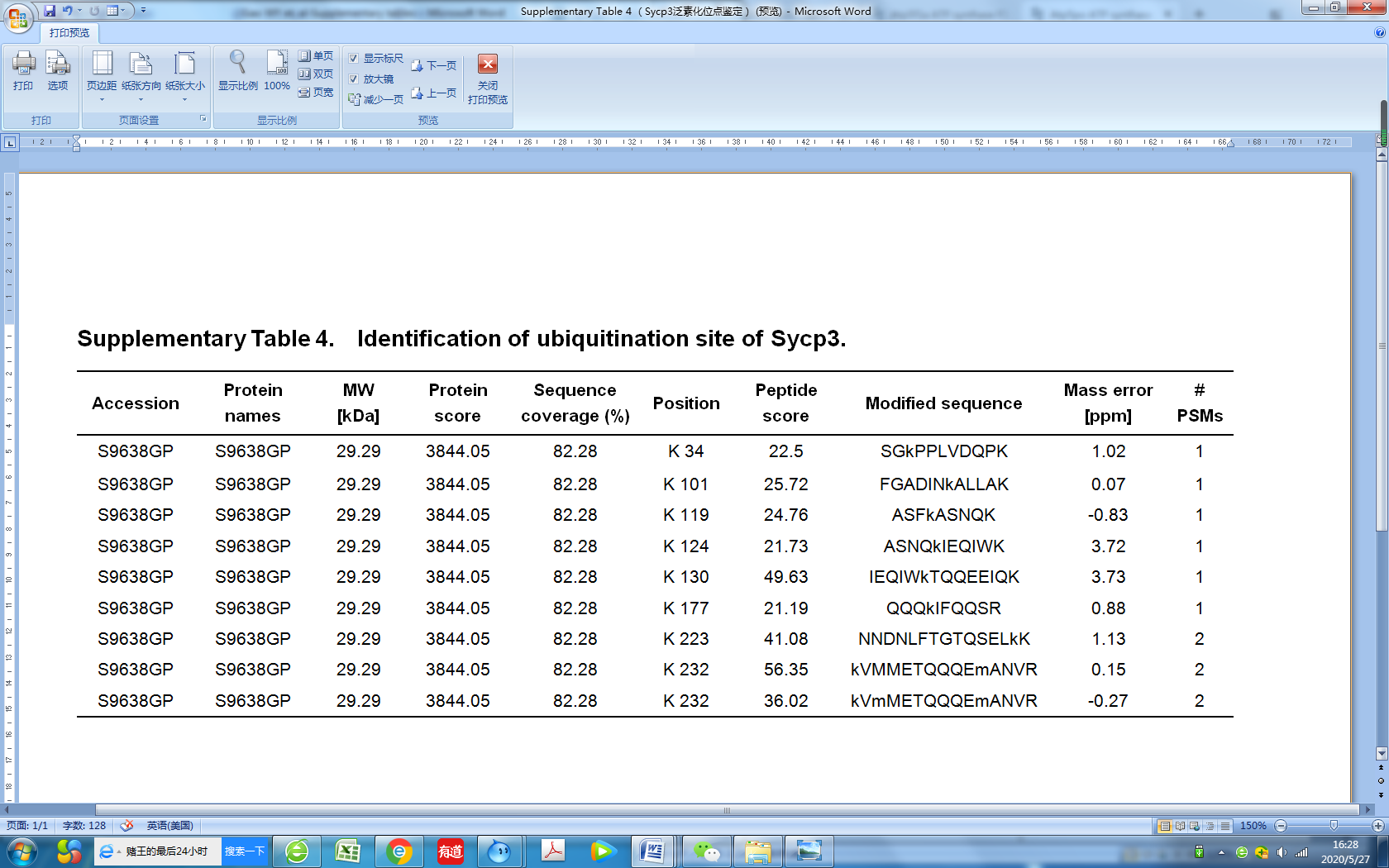
**Supplementary table 5. Identification of ubiquitination site in SYCP3 through mass spec.**

**Supplementary table 6. DNA templates for SiRNAs that were used for antibody validation**

**6.1. DNA o**ligos for SYCP3 siRNA

| **Target Site** | **DNA templates** |
| --- | --- |
| *Sycp3* CDS 542-562^1^ | Oligo1: GGATCCTAATACGACTCACTATAGAACTGGATATACAGAAATTT ^2^ |
|  | Oligo2: AAAAATTTCTGTATATCCAGTTCTATAGTGAGTCGTATTAGGATCC ^2^ |
|  | Oligo3: GGATCCTAATACGACTCACTATAAAATTTCTGTATATCCAGTTC^2^ |
|  | Oligo4: AAGAACTGGATATACAGAAATTTTATAGTGAGTCGTATTAGGATCC ^2^ |
| *Sycp3* CDS 597–618^1^ | Oligo1: GGATCCTAATACGACTCACTATAGACAACAACAAAAGATTTTTCA ^2^ |
|  | Oligo2: AATGAAAAATCTTTTGTTGTTGTC TATAGTGAGTCGTATTAGGATCC ^2^ |
|  | Oligo3: GGATCCTAATACGACTCACTATATGAAAAATCTTTTGTTGTTGTC ^2^ |
|  | Oligo4: AAGACAACAACAAAAGATTTTTCATATAGTGAGTCGTATTAGGATCC^2^ |
| *Sycp3* CDS 740–759^1^ | Oligo1: GGATCCTAATACGACTCACTATAGAACTTAAAAAAGAAATGGCT^2^ |
|  | Oligo2: AAAGCCATTTCTTTTTTAAGTTCTATAGTGAGTCGTATTAGGATCC^2^ |
|  | Oligo3: GGATCCTAATACGACTCACTATAAGCCATTTCTTTTTTAAGTTC^2^ |
|  | Oligo4: AAGAACTTAAAAAAGAAATGGCTTATAGTGAGTCGTATTAGGATCC ^2^ |
| Control^3^ | Oligo1: GGATCCTAATACGACTCACTATAGACCTACGCCACCAATTTCGT^2^ |
|  | Oligo2: AAACGAAATTGGTGGCGTAGGTCTATAGTGAGTCGTATTAGGATCC^2^ |
|  | Oligo3: GGATCCTAATACGACTCACTATAACGAAATTGGTGGCGTAGGTC^2^ |
|  | Oligo4: AAGACCTACGCCACCAATTTCGTTATAGTGAGTCGTATTAGGATCC ^2^ |

^1^ The numbers are the starting and ending position of the target sites in *Sycp3* CDS (NM_011517.2 in NCBI).

^2^ two pairs of DNA oligos are needed for for each double-stand siRNA. Oligo 2 is complementary with oligo 1 except an "AA" overhang at 5'; Oligo 3 is complementary with oligo 4 except an "AA" overhang at 5'. In each oligo, gene-specific sequences are underlined, other sequences are for recognition and binding by T7 RNA polymerase.

^3^ Control siRNA does not target to any mRNA sequence in mouse.

**6.2. DNA oligos for H2AX** siRNA

| **Target Site** | **DNA templates** |
| --- | --- |
| *H2ax*  CDS 184-205^1^ | Oligo1: GGATCCTAATACGACTCACTATAGAGATCCTGGAGCTGGCGGGCA^2^ |
|  | Oligo2: AATGCCCGCCAGCTCCAGGATCTCTATAGTGAGTCGTATTAGGATCC^2^ |
|  | Oligo3: GGATCCTAATACGACTCACTATATGCCCGCCAGCTCCAGGATCTC^2^ |
|  | Oligo4: AAGAGATCCTGGAGCTGGCGGGCATATAGTGAGTCGTATTAGGATCC^2^ |
| *H2ax*  CDS 217–238^1^ | Oligo1: GGATCCTAATACGACTCACTATAGACAACAAGAAGACGCGCATCA^2^ |
|  | Oligo2: AATGATGCGCGTCTTCTTGTTGTCTATAGTGAGTCGTATTAGGATCC^2^ |
|  | Oligo3: GGATCCTAATACGACTCACTATATGATGCGCGTCTTCTTGTTGTC^2^ |
|  | Oligo4:AAGACAACAAGAAGACGCGCATCATATAGTGAGTCGTATTAGGATCC^2^ |
| *H2ax*  CDS 228–248^1^ | Oligo1: GGATCCTAATACGACTCACTATAGACGCGCATCATCCCGCGCCA^2^ |
|  | Oligo2: AATGGCGCGGGATGATGCGCGTCTATAGTGAGTCGTATTAGGATCC^2^ |
|  | Oligo3: GGATCCTAATACGACTCACTATATGGCGCGGGATGATGCGCGTC^2^ |
|  | Oligo4:AAGACGCGCATCATCCCGCGCCATATAGTGAGTCGTATTAGGATCC^2^ |
| Control^3^ | Oligo1: GGATCCTAATACGACTCACTATAGACCTACGCCACCAATTTCGT^2^ |
|  | Oligo2: AAACGAAATTGGTGGCGTAGGTCTATAGTGAGTCGTATTAGGATCC^2^ |
|  | Oligo3: GGATCCTAATACGACTCACTATAACGAAATTGGTGGCGTAGGTC^2^ |
|  | Oligo4: AAGACCTACGCCACCAATTTCGTTATAGTGAGTCGTATTAGGATCC ^2^ |

^1^ The numbers are the starting and ending position of the target sites in *H2ax* CDS (NM_010436.2 in NCBI).

^2^ two pairs of DNA oligos are needed for for each double-stand siRNA. Oligo 2 is complementary with oligo 1 except an "AA" overhang at 5'; Oligo 3 is complementary with oligo 4 except an "AA" overhang at 5'. In each oligo, gene-specific sequences are underlined, other sequences are for recognition and binding by T7 RNA polymerase.

^3^ Control siRNA does not target to any mRNA sequence in mouse.

**6.3. DNA oligos for RAD51** siRNA

| **Target Site** | **DNA templates** |
| --- | --- |
| *Rad51* CDS 867-888^1^ | Oligo1: GGATCCTAATACGACTCACTATAGAACATCATCGCTCATGCGTCA^2^ |
|  | Oligo2:AATGACGCATGAGCGATGATGTTCTATAGTGAGTCGTATTAGGATCC^2^ |
|  | Oligo3: GGATCCTAATACGACTCACTATATGACGCATGAGCGATGATGTTC^2^ |
|  | Oligo4: AAGAACATCATCGCTCATGCGTCATATAGTGAGTCGTATTAGGATCC^2^ |
| *Rad51* CDS 908–928^1^ | Oligo1: GGATCCTAATACGACTCACTATAGAAAAGGAAGAGGGGAGACCA^2^ |
|  | Oligo2: AATGGTCTCCCCTCTTCCTTTTCTATAGTGAGTCGTATTAGGATCC^2^ |
|  | Oligo3: GGATCCTAATACGACTCACTATATGGTCTCCCCTCTTCCTTTTC^2^ |
|  | Oligo4:AAGAAAAGGAAGAGGGGAGACCATATAGTGAGTCGTATTAGGATCC^2^ |
| *Rad51* CDS 929–949^1^ | Oligo1: GGATCCTAATACGACTCACTATAGAATCTGCAAAATCTATGACT^2^ |
|  | Oligo2: AAAGTCATAGATTTTGCAGATTCTATAGTGAGTCGTATTAGGATCC^2^ |
|  | Oligo3: GGATCCTAATACGACTCACTATAAGTCATAGATTTTGCAGATTC^2^ |
|  | Oligo4:AAGAATCTGCAAAATCTATGACTTATAGTGAGTCGTATTAGGATCC^2^ |
| Control^3^ | Oligo1: GGATCCTAATACGACTCACTATAGACCTACGCCACCAATTTCGT^2^ |
|  | Oligo2: AAACGAAATTGGTGGCGTAGGTCTATAGTGAGTCGTATTAGGATCC^2^ |
|  | Oligo3: GGATCCTAATACGACTCACTATAACGAAATTGGTGGCGTAGGTC^2^ |
|  | Oligo4: AAGACCTACGCCACCAATTTCGTTATAGTGAGTCGTATTAGGATCC ^2^ |

^1^ The numbers are the starting and ending position of the target sites in *Rad51* CDS (NM_011234.5 in NCBI).

^2^ two pairs of DNA oligos are needed for for each double-stand siRNA. Oligo 2 is complementary with oligo 1 except an "AA" overhang at 5'; Oligo 3 is complementary with oligo 4 except an "AA" overhang at 5'. In each oligo, gene-specific sequences are underlined, other sequences are for recognition and binding by T7 RNA polymerase.

^3^ Control siRNA does not target to any mRNA sequence in mouse.

**6.4. DNA oligos for CHK2 siRNA**

| **Target Site** | **DNA templates** |
| --- | --- |
| *Chk2*  CDS 651-672^1^ | Oligo1: GGATCCTAATACGACTCACTATAGACAGATGTCTCAGAGGAGGCT^2^ |
|  | Oligo2: AAAGCCTCCTCTGAGACATCTGTCTATAGTGAGTCGTATTAGGATCC^2^ |
|  | Oligo3: GGATCCTAATACGACTCACTATAAGCCTCCTCTGAGACATCTGTC^2^ |
|  | Oligo4: AAGACAGATGTCTCAGAGGAGGCTTATAGTGAGTCGTATTAGGATCC^2^ |
| *Chk2*  CDS 676–696^1^ | Oligo1: GGATCCTAATACGACTCACTATAGACCTTGTCAAGAAACTGTTA^2^ |
|  | Oligo2: AATAACAGTTTCTTGACAAGGTCTATAGTGAGTCGTATTAGGATCC^2^ |
|  | Oligo3: GGATCCTAATACGACTCACTATATAACAGTTTCTTGACAAGGTC^2^ |
|  | Oligo4: AAGACCTTGTCAAGAAACTGTTATATAGTGAGTCGTATTAGGATCC^2^ |
| *Chk2*  CDS 703–724^1^ | Oligo1: GGATCCTAATACGACTCACTATAGACCCAAAGGCTCGGCTTACCA^2^ |
|  | Oligo2: AATGGTAAGCCGAGCCTTTGGGTCTATAGTGAGTCGTATTAGGATCC^2^ |
|  | Oligo3: GGATCCTAATACGACTCACTATATGGTAAGCCGAGCCTTTGGGTC^2^ |
|  | Oligo4: AAGACCCAAAGGCTCGGCTTACCATATAGTGAGTCGTATTAGGATCC^2^ |
| Control^3^ | Oligo1: GGATCCTAATACGACTCACTATAGACCTACGCCACCAATTTCGT^2^ |
|  | Oligo2: AAACGAAATTGGTGGCGTAGGTCTATAGTGAGTCGTATTAGGATCC^2^ |
|  | Oligo3: GGATCCTAATACGACTCACTATAACGAAATTGGTGGCGTAGGTC^2^ |
|  | Oligo4: AAGACCTACGCCACCAATTTCGTTATAGTGAGTCGTATTAGGATCC ^2^ |

^1^ The numbers are the starting and ending position of the target sites in *Chk2* CDS (NM_001363308.1 in NCBI).

^2^ two pairs of DNA oligos are needed for for each double-stand siRNA. Oligo 2 is complementary with oligo 1 except an "AA" overhang at 5'; Oligo 3 is complementary with oligo 4 except an "AA" overhang at 5'. In each oligo, gene-specific sequences are underlined, other sequences are for recognition and binding by T7 RNA polymerase.

^3^ Control siRNA does not target to any mRNA sequence in mouse.

**6.5. DNA oligos for STAT1** siRNA

| **Target Site** | **DNA templates** |
| --- | --- |
| *Stat1* CDS 1207-1228^1^ | Oligo1: GGATCCTAATACGACTCACTATAGAGTTCCGACACCTGCAACTGA ^2^ |
|  | Oligo2: AATCAGTTGCAGGTGTCGGAACTCTATAGTGAGTCGTATTAGGATCC ^2^ |
|  | Oligo3: GGATCCTAATACGACTCACTATATCAGTTGCAGGTGTCGGAACTC^2^ |
|  | Oligo4: AAGAGTTCCGACACCTGCAACTGATATAGTGAGTCGTATTAGGATCC^2^ |
| *Stat1* CDS 1285–1305^1^ | Oligo1: GGATCCTAATACGACTCACTATAGAACTTCACTCTCTTAGCTTT ^2^ |
|  | Oligo2: AAAAAGCTAAGAGAGTGAAGTTCTATAGTGAGTCGTATTAGGATCC ^2^ |
|  | Oligo3: GGATCCTAATACGACTCACTATAAAAGCTAAGAGAGTGAAGTTC^2^ |
|  | Oligo4: AAGAACTTCACTCTCTTAGCTTTTATAGTGAGTCGTATTAGGATCC^2^ |
| *Stat1* CDS 1456–1477^1^ | Oligo1: GGATCCTAATACGACTCACTATAGAGCCCAGGAATCTCTCCTTCT^2^ |
|  | Oligo2: AAAGAAGGAGAGATTCCTGGGCTCTATAGTGAGTCGTATTAGGATCC^2^ |
|  | Oligo3: GGATCCTAATACGACTCACTATAAGAAGGAGAGATTCCTGGGCTC^2^ |
|  | Oligo4: AAGAGCCCAGGAATCTCTCCTTCTTATAGTGAGTCGTATTAGGATCC^2^ |
| Control^3^ | Oligo1: GGATCCTAATACGACTCACTATAGACCTACGCCACCAATTTCGT^2^ |
|  | Oligo2: AAACGAAATTGGTGGCGTAGGTCTATAGTGAGTCGTATTAGGATCC^2^ |
|  | Oligo3: GGATCCTAATACGACTCACTATAACGAAATTGGTGGCGTAGGTC^2^ |
|  | Oligo4: AAGACCTACGCCACCAATTTCGTTATAGTGAGTCGTATTAGGATCC ^2^ |

^1^ The numbers are the starting and ending position of the target sites in *Stat1* CDS (NM_001205313.1 in NCBI).

^2^ two pairs of DNA oligos are needed for for each double-stand siRNA. Oligo 2 is complementary with oligo 1 except an "AA" overhang at 5'; Oligo 3 is complementary with oligo 4 except an "AA" overhang at 5'. In each oligo, gene-specific sequences are underlined, other sequences are for recognition and binding by T7 RNA polymerase.

^3^ Control siRNA does not target to any mRNA sequence in mouse.

**6.6. DNA oligos for DTX3L** siRNA

| **Target Site** | **DNA templates** |
| --- | --- |
| *Dtx3l* CDS 2033-2054^1^ | Oligo1: GGATCCTAATACGACTCACTATAGAAGAAAGGTTTTGGATCTGCT^2^ |
|  | Oligo2: AAAGCAGATCCAAAACCTTTCTTCTATAGTGAGTCGTATTAGGATCC^2^ |
|  | Oligo3: GGATCCTAATACGACTCACTATAAGCAGATCCAAAACCTTTCTTC^2^ |
|  | Oligo4: AAGAAGAAAGGTTTTGGATCTGCTTATAGTGAGTCGTATTAGGATCC^2^ |
| *Dtx3l* CDS 2075–2095^1^ | Oligo1: GGATCCTAATACGACTCACTATAGACTGACTTTCACAATAGGAT^2^ |
|  | Oligo2: AAATCCTATTGTGAAAGTCAGTCTATAGTGAGTCGTATTAGGATCC^2^ |
|  | Oligo3: GGATCCTAATACGACTCACTATAATCCTATTGTGAAAGTCAGTC^2^ |
|  | Oligo4: AAGACTGACTTTCACAATAGGATTATAGTGAGTCGTATTAGGATCC^2^ |
| *Dtx3l* CDS 2119–2139^1^ | Oligo1: GGATCCTAATACGACTCACTATAGATGTCATTACATGGAATGAT^2^ |
|  | Oligo2: AAATCATTCCATGTAATGACATCTATAGTGAGTCGTATTAGGATCC^2^ |
|  | Oligo3: GGATCCTAATACGACTCACTATAATCATTCCATGTAATGACATC^2^ |
|  | Oligo4: AAGATGTCATTACATGGAATGATTATAGTGAGTCGTATTAGGATCC^2^ |
| Control^3^ | Oligo1: GGATCCTAATACGACTCACTATAGACCTACGCCACCAATTTCGT^2^ |
|  | Oligo2: AAACGAAATTGGTGGCGTAGGTCTATAGTGAGTCGTATTAGGATCC^2^ |
|  | Oligo3: GGATCCTAATACGACTCACTATAACGAAATTGGTGGCGTAGGTC^2^ |
|  | Oligo4: AAGACCTACGCCACCAATTTCGTTATAGTGAGTCGTATTAGGATCC ^2^ |

^1^ The numbers are the starting and ending position of the target sites in *Dtx3l* CDS (NM_001013371.2 in NCBI).

^2^ two pairs of DNA oligos are needed for for each double-stand siRNA. Oligo 2 is complementary with oligo 1 except an "AA" overhang at 5'; Oligo 3 is complementary with oligo 4 except an "AA" overhang at 5'. In each oligo, gene-specific sequences are underlined, other sequences are for recognition and binding by T7 RNA polymerase.

^3^ Control siRNA does not target to any mRNA sequence in mouse.
