# Supplementary figures and images for: FBXW24 controls female meiotic prophase progression by regulating SYCP3 ubiquitination
Source: Clin Transl Med. 2022 Jul 20;12(7):e891. doi: 10.1002/ctm2.891 (PMC9299759; doi:10.1002/ctm2.891)

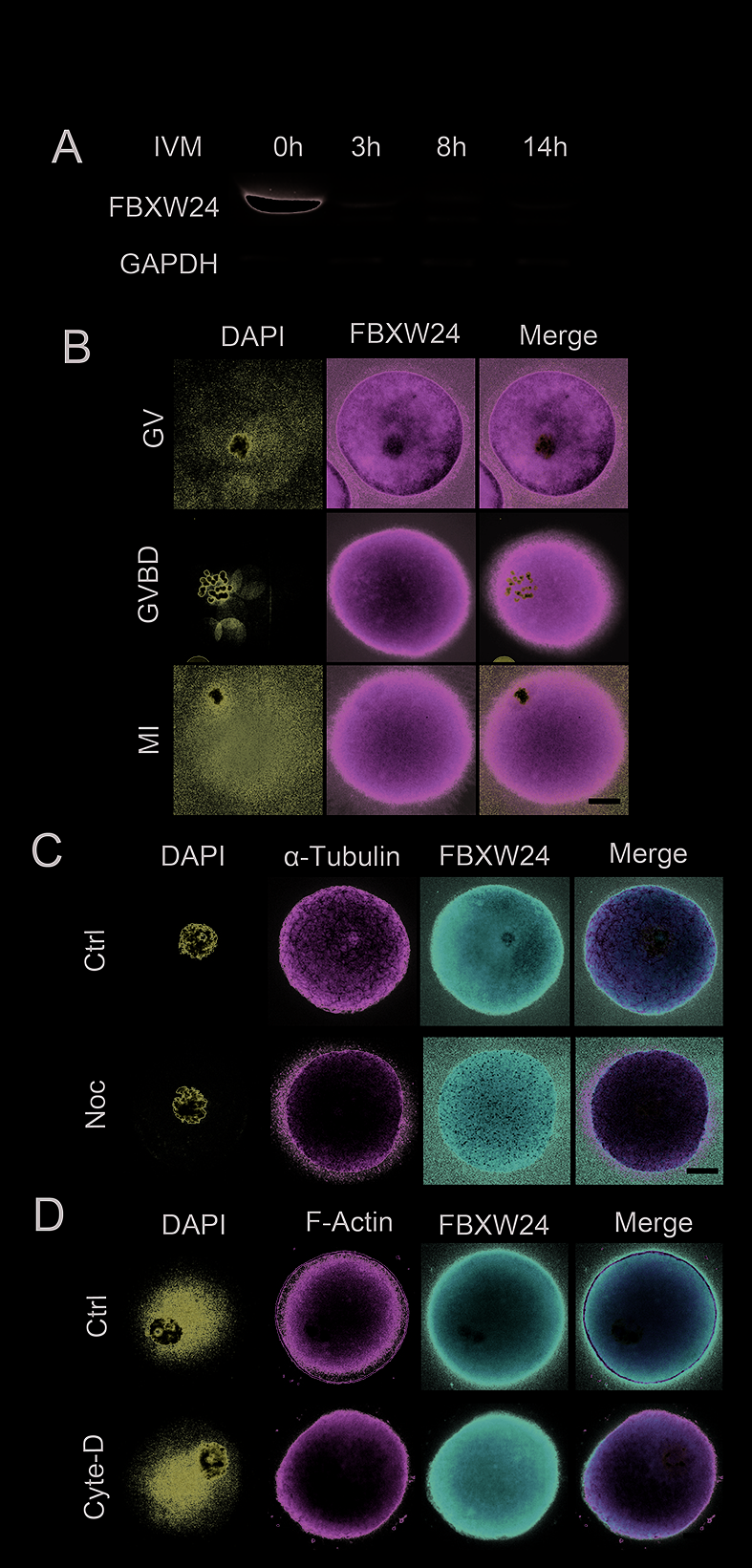

Supplement: Supplementary file 4 — Supporting Information [file CTM2-12-e891-s008.tif]

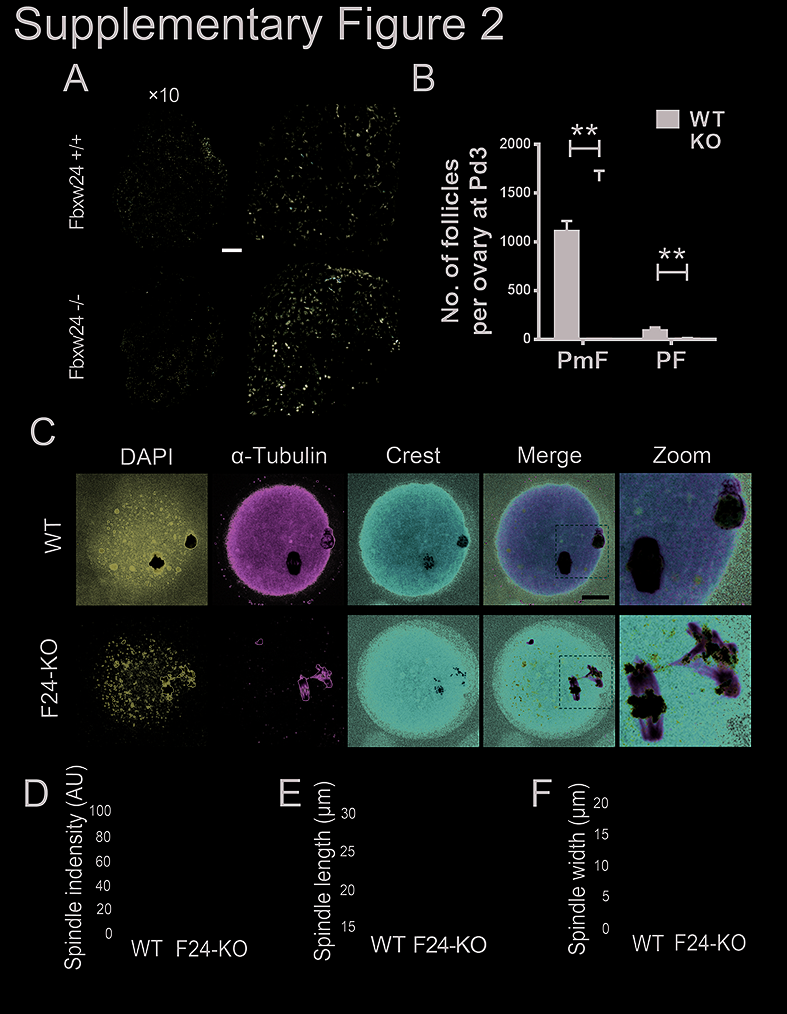

Supplement: Supplementary file 5 — Supporting Information [file CTM2-12-e891-s022.tif]

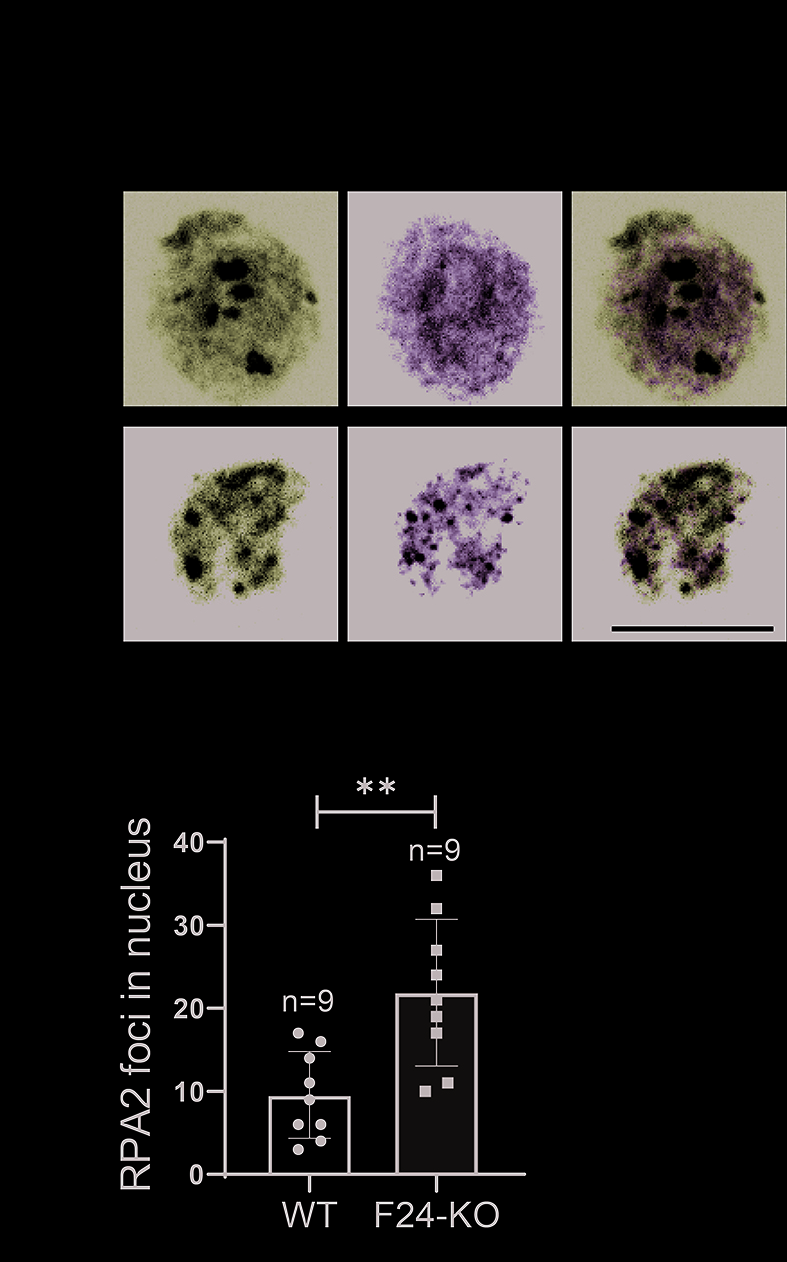

Supplement: Supplementary file 6 — Supporting Information [file CTM2-12-e891-s018.tif]

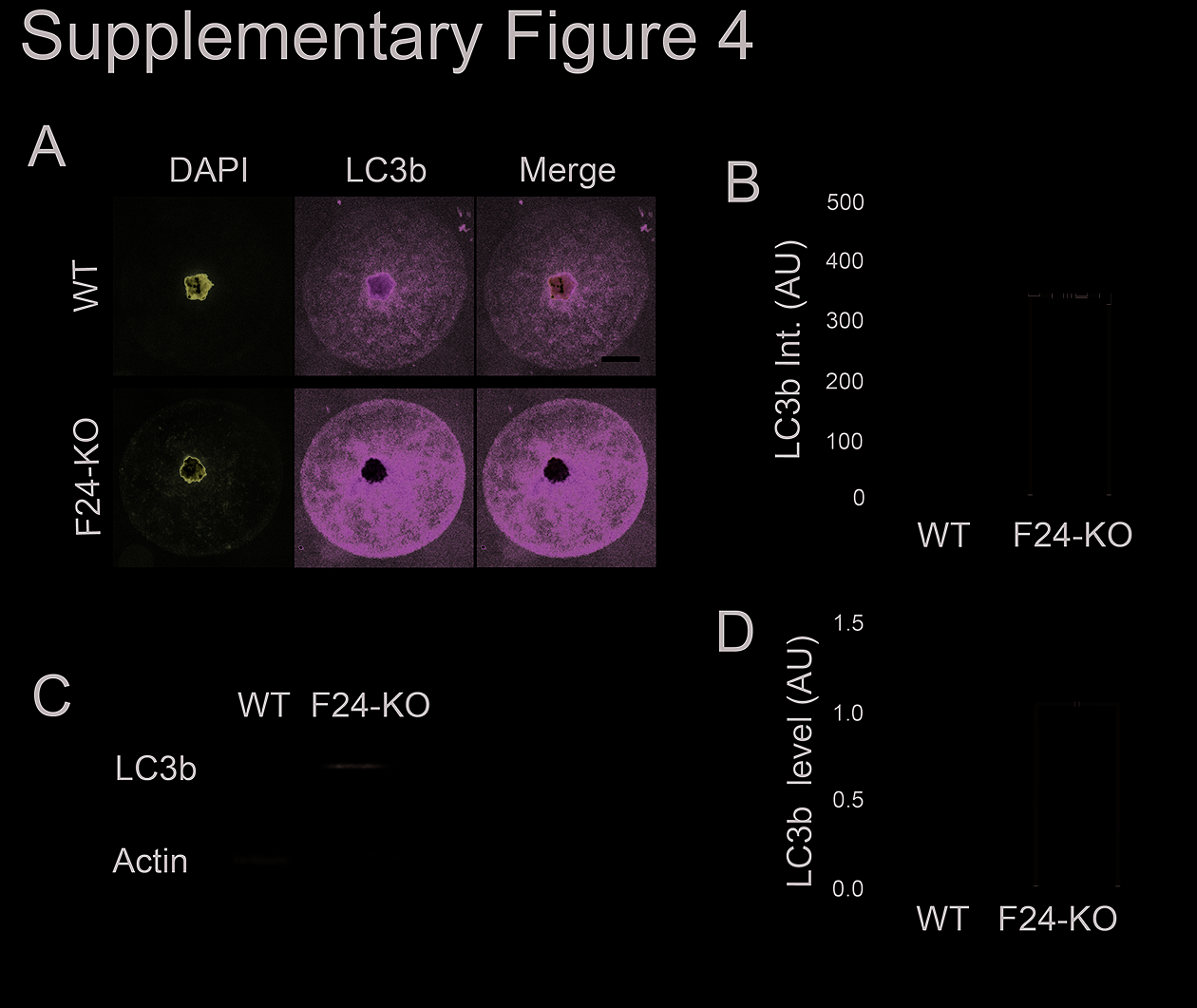

Supplement: Supplementary file 7 — Supporting Information [file CTM2-12-e891-s016.tif]

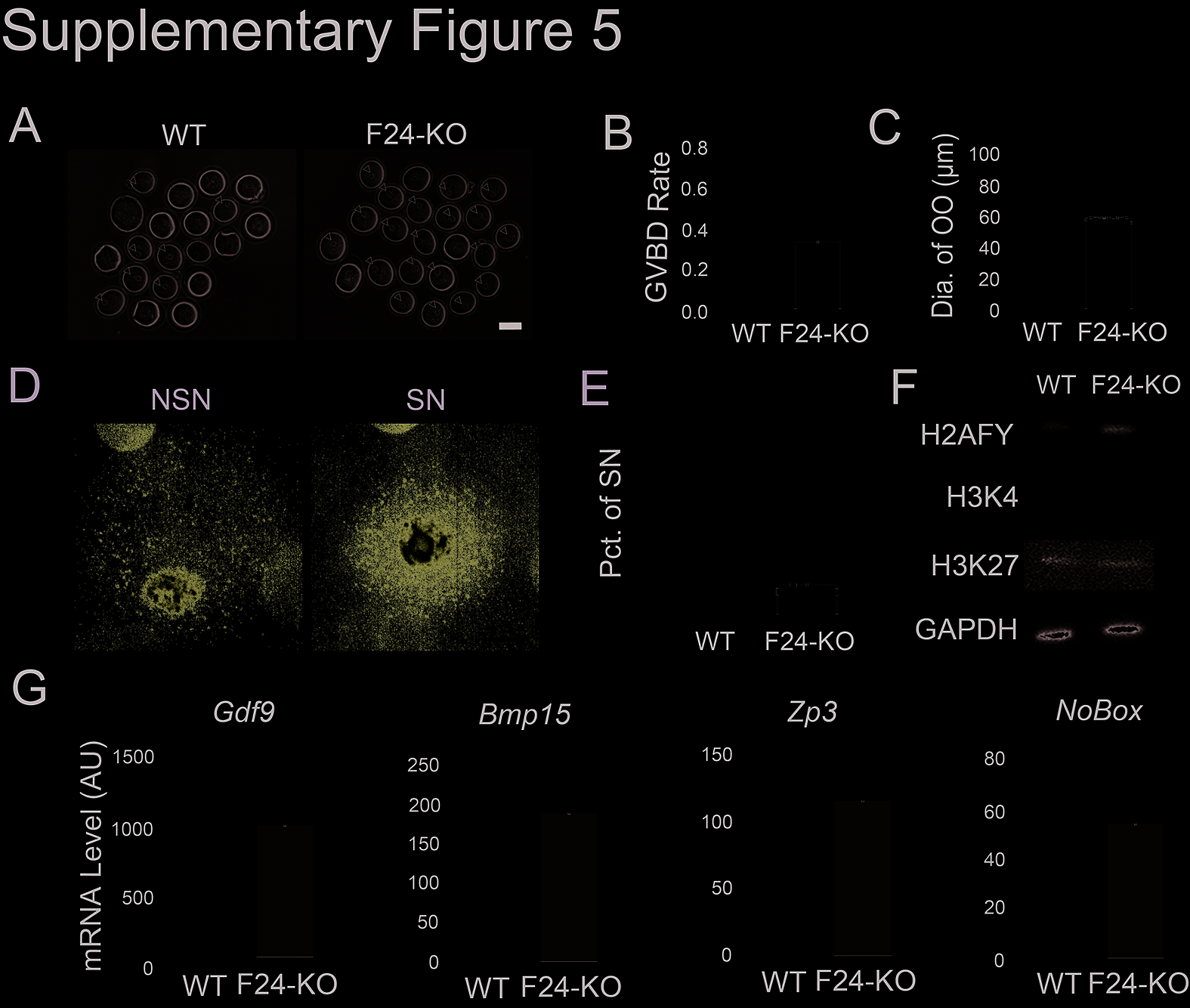

Supplement: Supplementary file 8 — Supporting Information [file CTM2-12-e891-s023.tif]

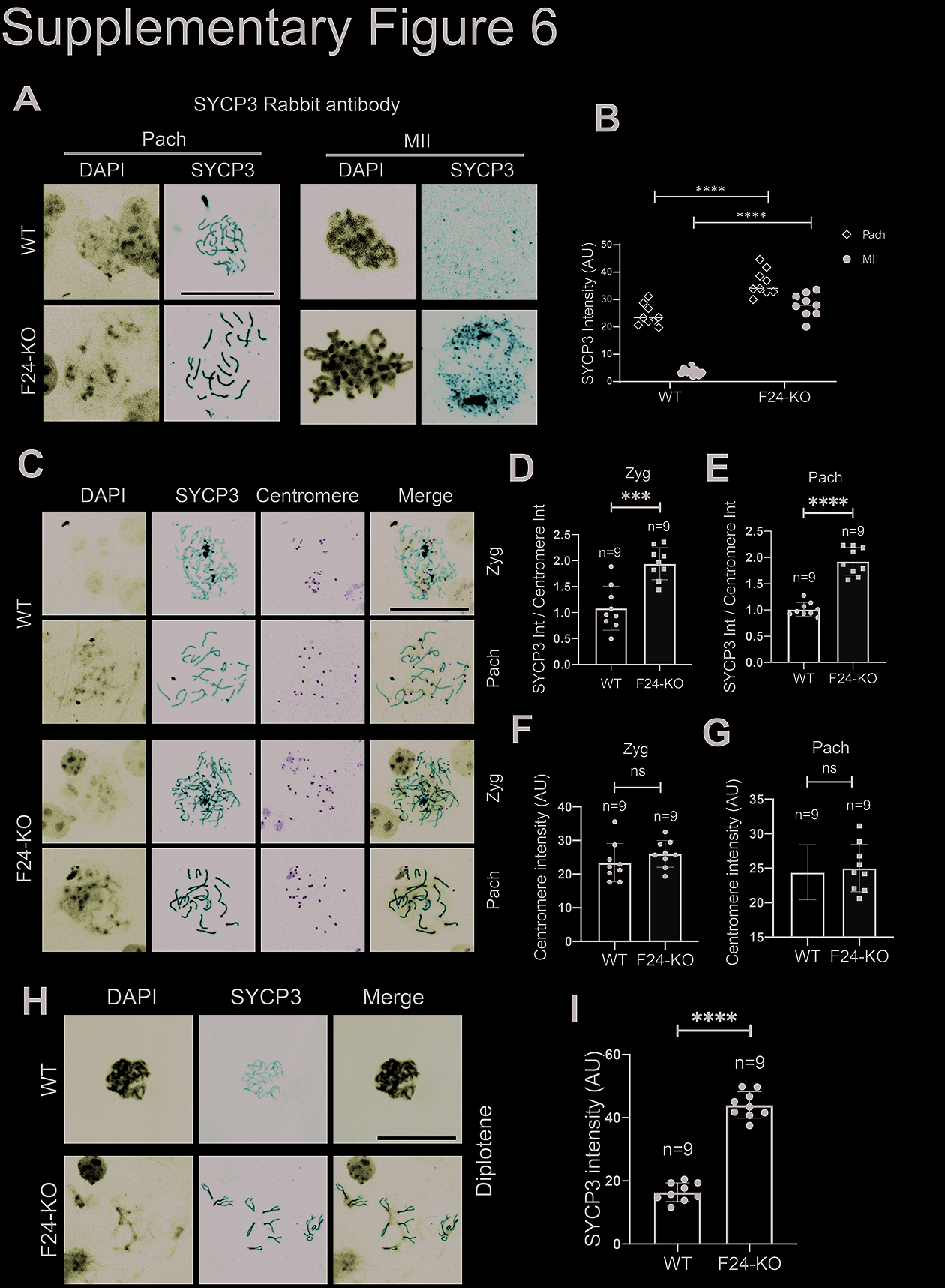

Supplement: Supplementary file 9 — Supporting Information [file CTM2-12-e891-s009.tif]

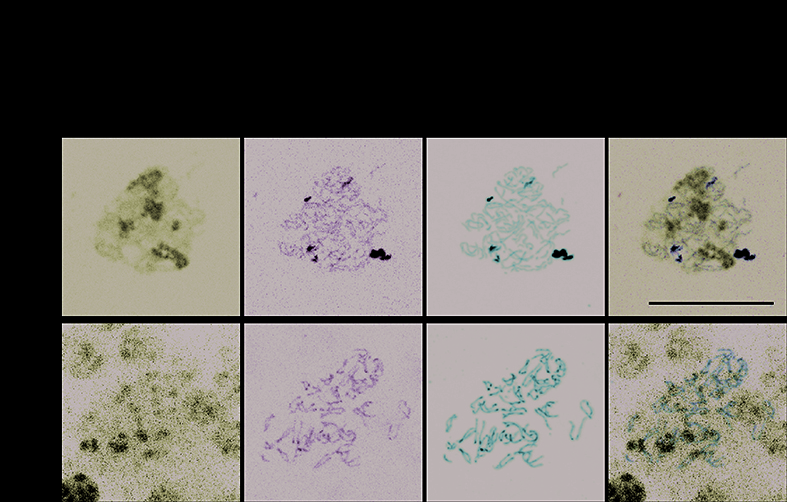

Supplement: Supplementary file 10 — Supporting Information [file CTM2-12-e891-s005.tif]

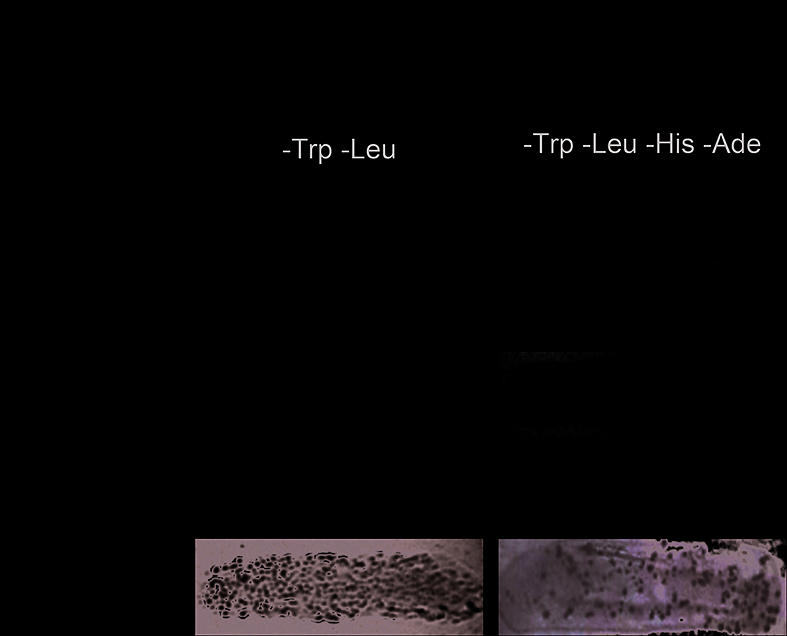

Supplement: Supplementary file 11 — Supporting Information [file CTM2-12-e891-s025.tif]

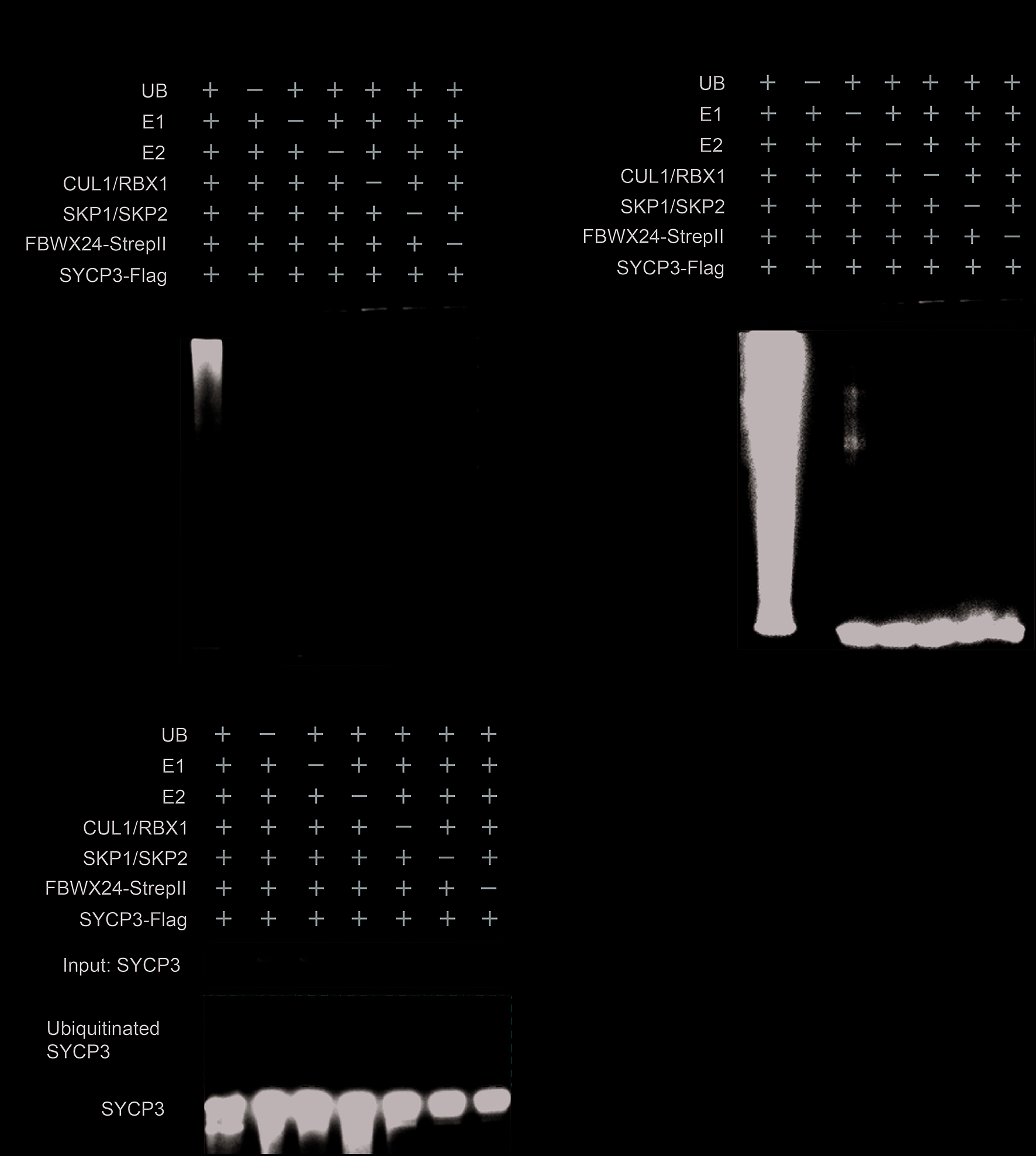

Supplement: Supplementary file 12 — Supporting Information [file CTM2-12-e891-s010.tif]

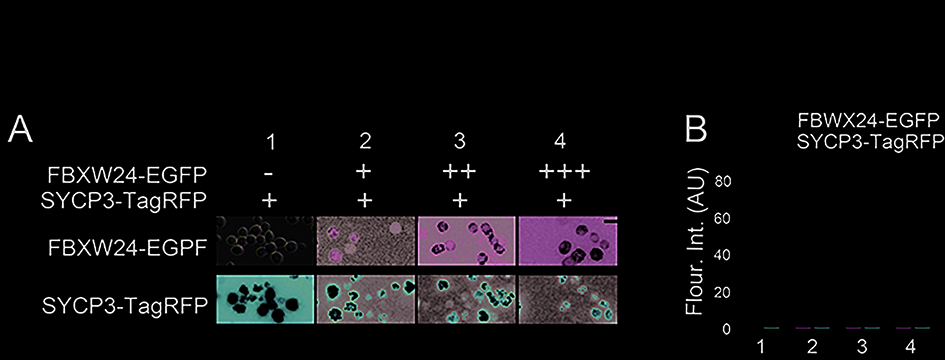

Supplement: Supplementary file 13 — Supporting Information [file CTM2-12-e891-s015.tif]

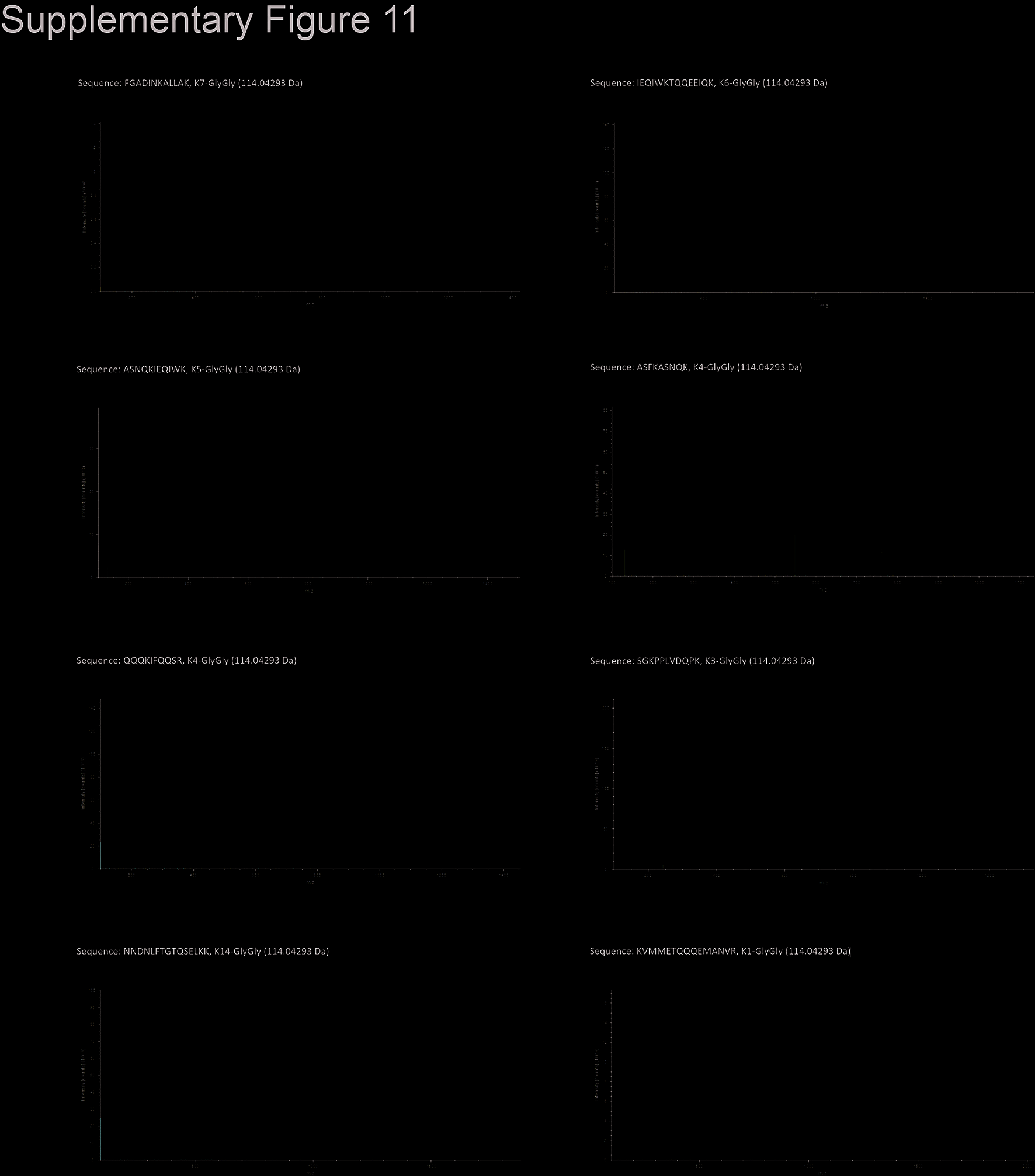

Supplement: Supplementary file 14 — Supporting Information [file CTM2-12-e891-s021.tif]

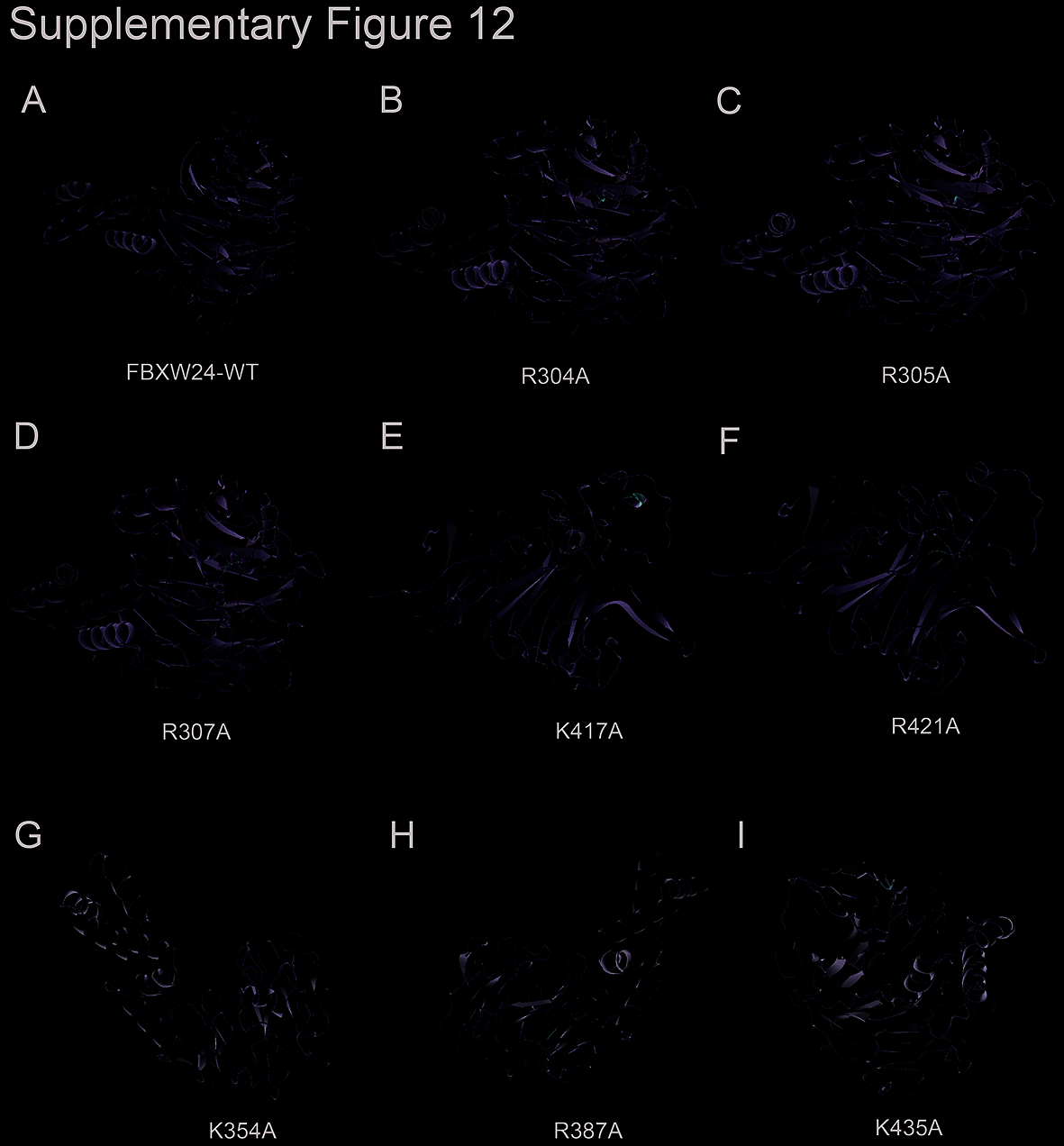

Supplement: Supplementary file 15 — Supporting Information [file CTM2-12-e891-s006.tif]

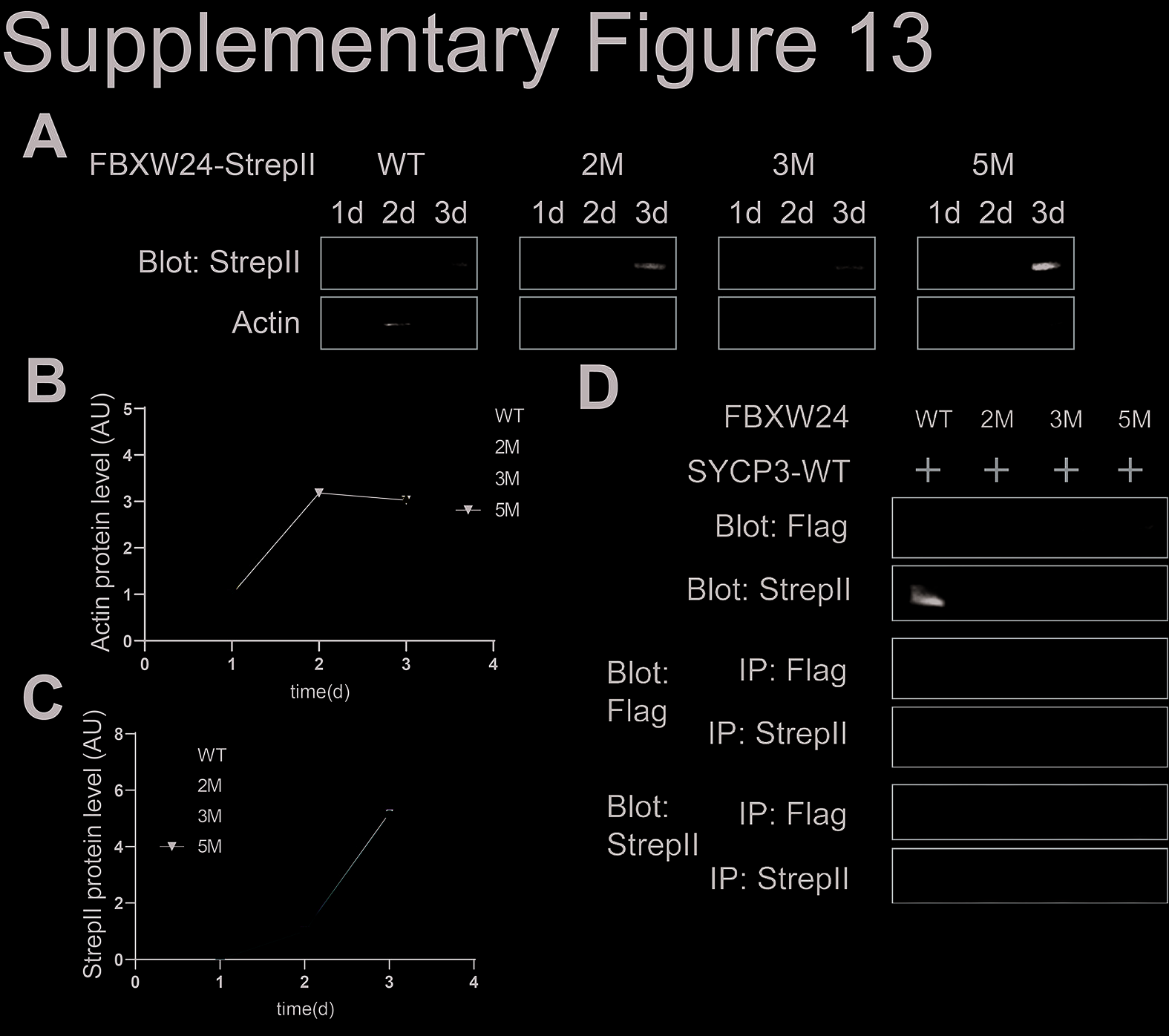

Supplement: Supplementary file 16 — Supporting Information [file CTM2-12-e891-s003.tif]

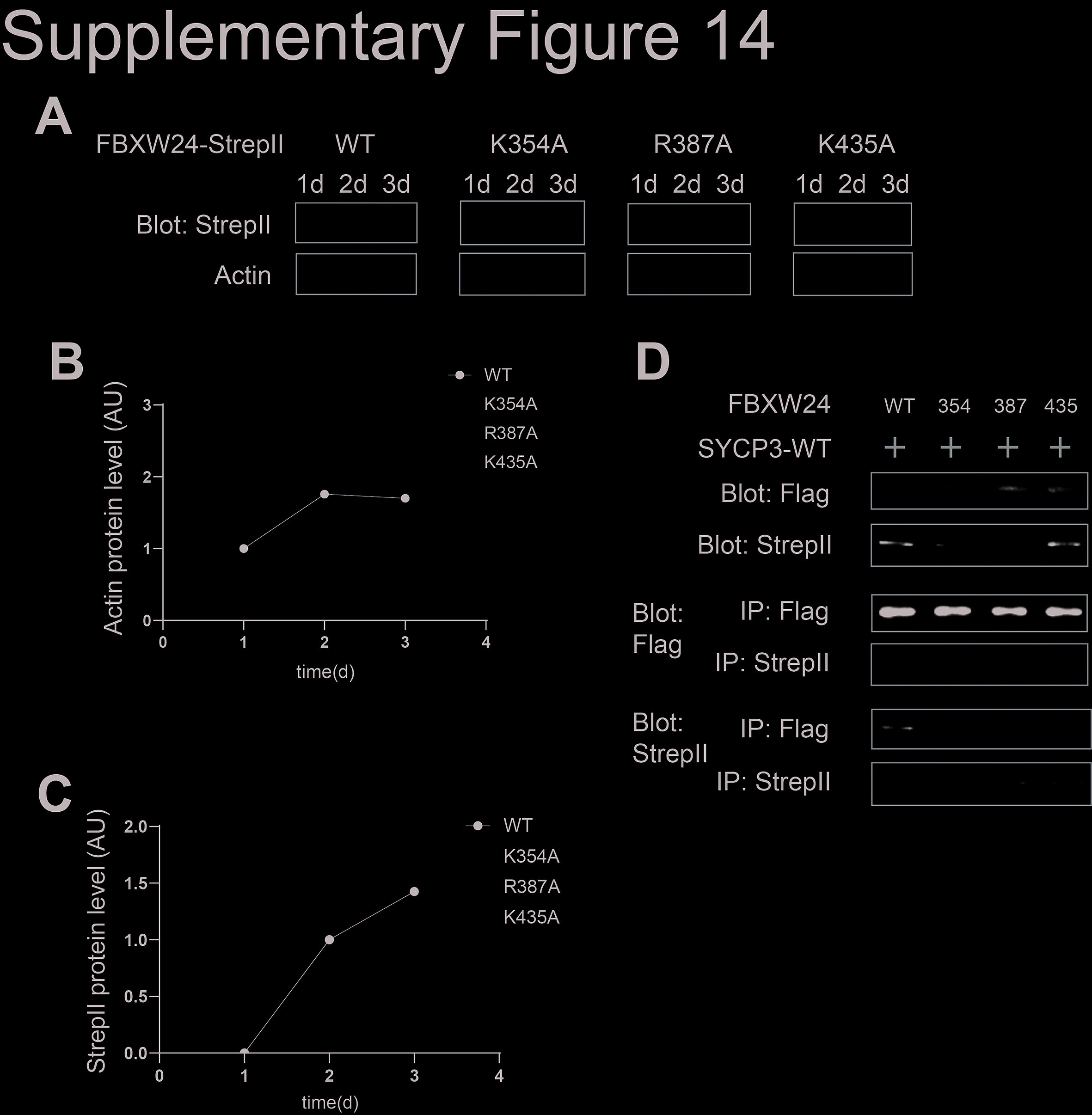

Supplement: Supplementary file 17 — Supporting Information [file CTM2-12-e891-s001.tif]

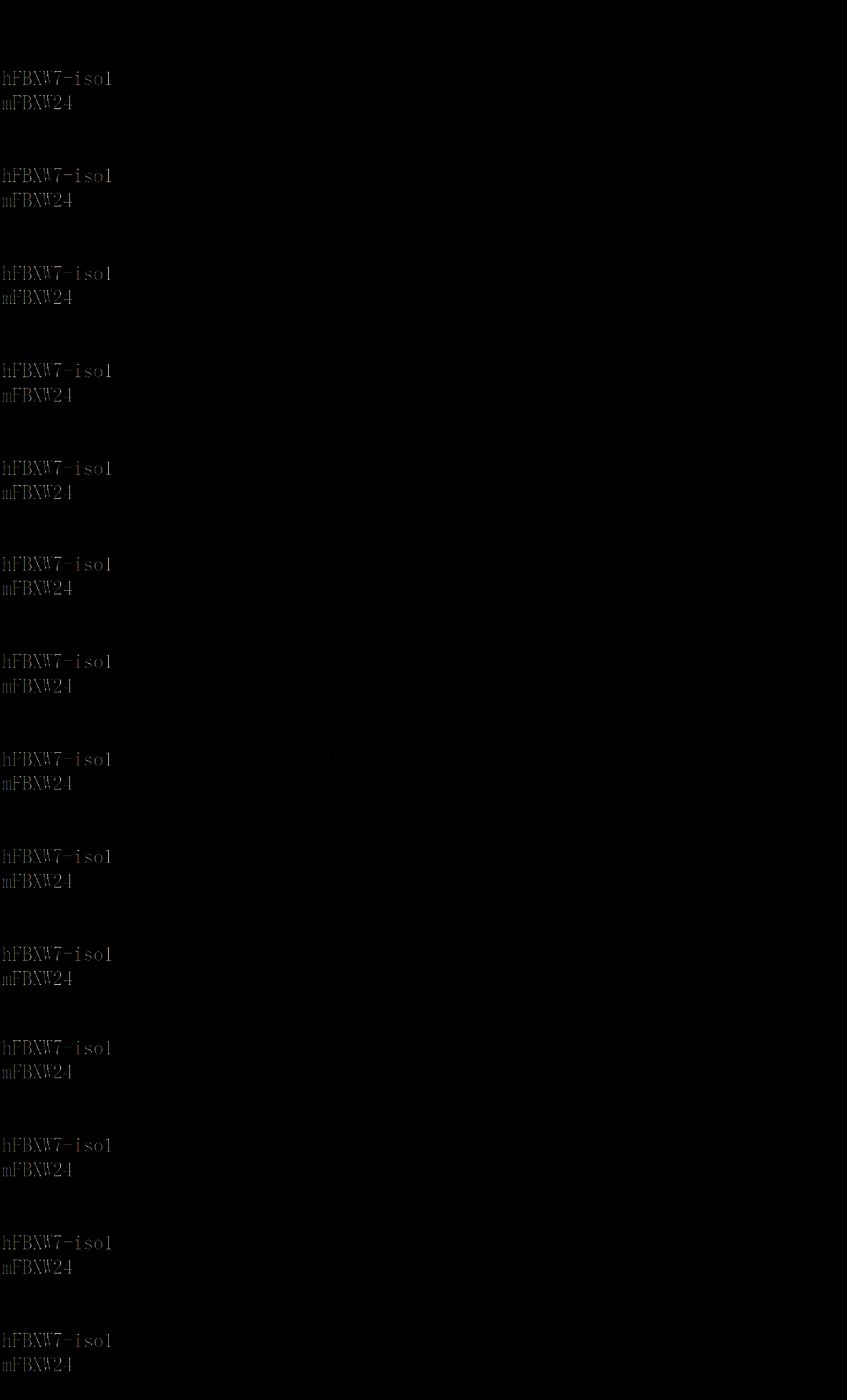

Supplement: Supplementary file 18 — Supporting Information [file CTM2-12-e891-s013.tif]

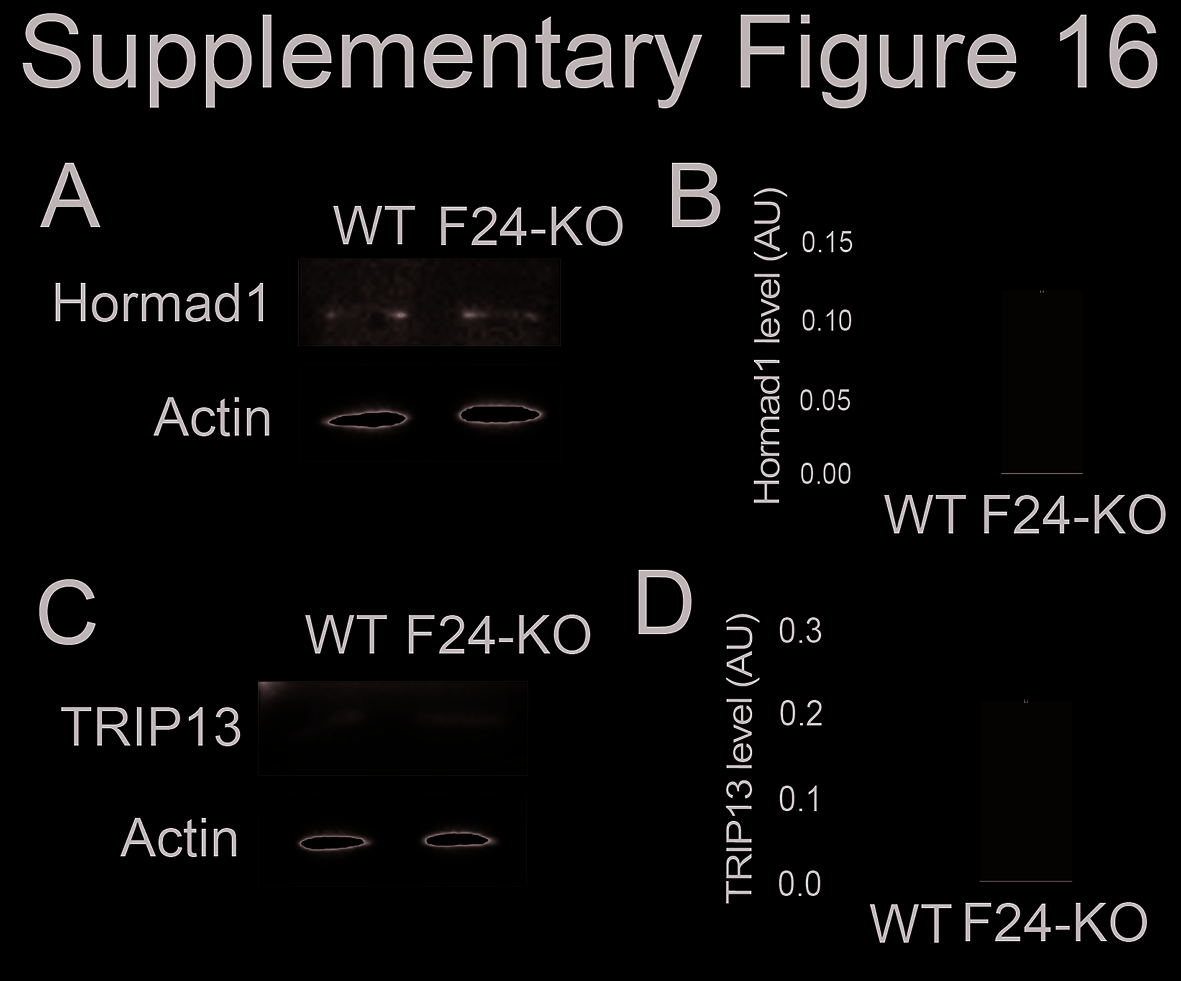

Supplement: Supplementary file 19 — Supporting Information [file CTM2-12-e891-s017.tif]

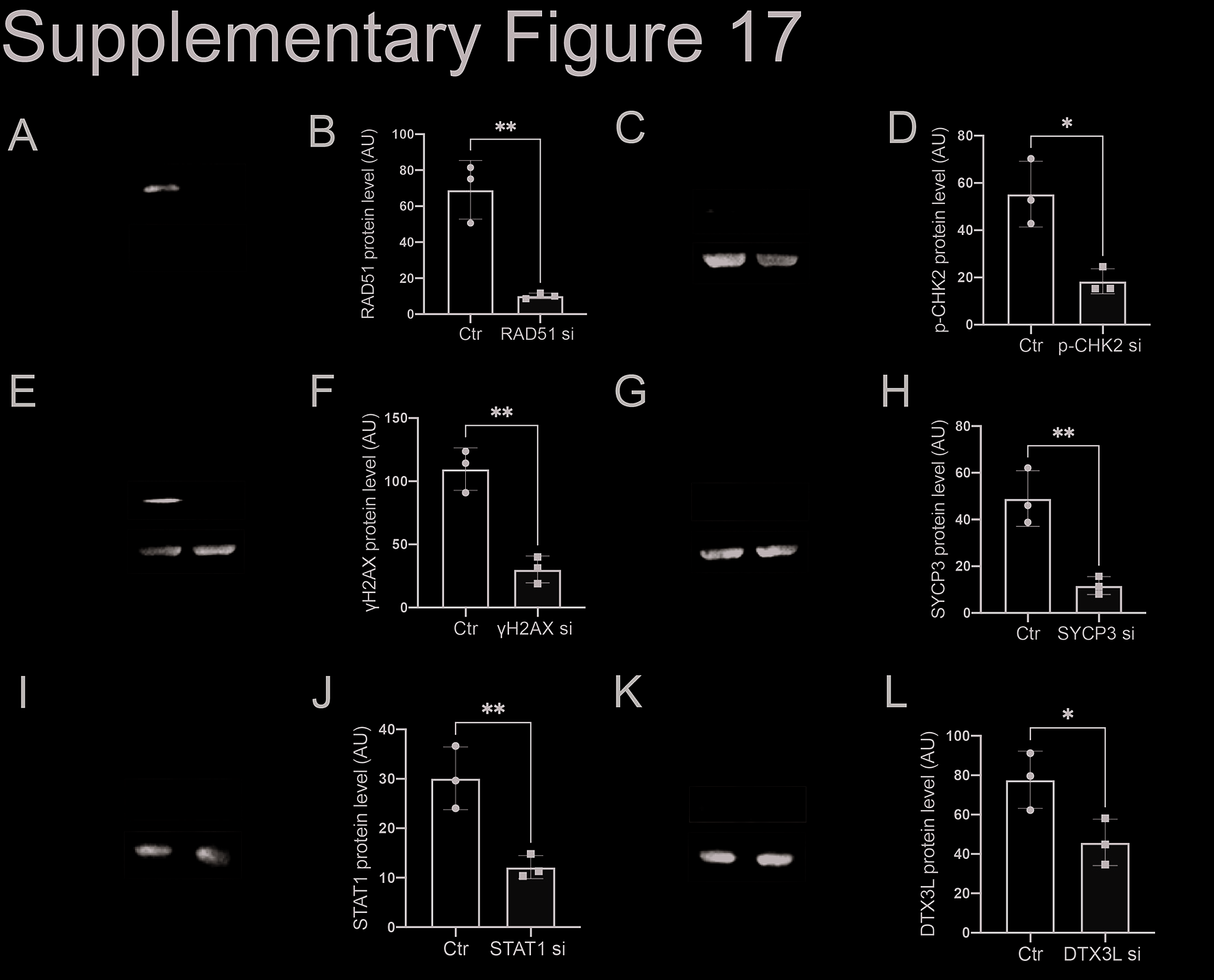

Supplement: Supplementary file 20 — Supporting Information [file CTM2-12-e891-s024.tif]
